# Improving the genetic system for *Halorubrum lacusprofundi* to allow in-frame deletions

**DOI:** 10.3389/fmicb.2023.1095621

**Published:** 2023-03-31

**Authors:** L. Johanna Gebhard, Iain G. Duggin, Susanne Erdmann

**Affiliations:** ^1^Archaeal Virology, Max Planck Institute for Marine Microbiology, Bremen, Germany; ^2^The Australian Institute for Microbiology and Infection, University of Technology Sydney, Sydney, NSW, Australia

**Keywords:** *Halorubrum lacusprofundi*, archaea, genetic system, cold adaptation, haloarchaea, auxotrophic mutant

## Abstract

*Halorubrum lacusprofundi* is a cold-adapted halophilic archaeon isolated from Deep Lake, Antarctica. *Hrr. lacusprofundi* is commonly used to study adaptation to cold environments and thereby a potential source for biotechnological products. Additionally, in contrast to other haloarchaeal model organisms, *Hrr. lacusprofundi* is also susceptible to a range of different viruses and virus-like elements, making it a great model to study virus-host interactions in a cold-adapted organism. A genetic system has previously been reported for *Hrr. lacusprofundi*; however, it does not allow in-frame deletions and multiple gene knockouts. Here, we report the successful generation of uracil auxotrophic (*pyrE2*) mutants of two strains of *Hrr. lacusprofundi*. Subsequently, we attempted to generate knockout mutants using the auxotrophic marker for selection. However, surprisingly, only the combination of the auxotrophic marker and antibiotic selection allowed the timely and clean in-frame deletion of a target gene. Finally, we show that vectors established for the model organism *Haloferax volcanii* are deployable for genetic manipulation of *Hrr. lacusprofundi*, allowing the use of the portfolio of genetic tools available for *H. volcanii* in *Hrr. lacusprofundi*.

## Introduction

Halophilic archaea (haloarchaea) are established as commonly used model systems to study archaeal cell biology and virus-host interactions in archaea (Luk et al., 2014; van Wolferen et al., 2022). Additionally, they are of great interest for their potential use in biotechnological applications (Litchfield, 2011; Javier et al., 2017; Singh and Singh, 2017; Correa and Abreu, 2020; Li et al., 2022). They are easy to culture and genetic systems have already been developed for some members, with *Haloferax volcanii* currently being the most studied model organism (Hartman et al., 2010; Pohlschroder and Schulze, 2019). However, only one virus has been isolated for *H. volcanii* so far (Alarcón-Schumacher et al., 2022), while the majority of hosts for haloarchaeal viruses remain genetically inaccessible. In contrast to *Haloferax* species, that have been shown to be rather unsusceptible to virus infection (Aguirre Sourrouille et al., 2022), a great number of haloarchaeal viruses have been isolated on *Halorubrum* species (Atanasova et al., 2015; Pietilä et al., 2016; Liu et al., 2021). *Halorubrum lacusprofundi* ATCC 49239 (ACAM34) (Franzmann et al., 1988; McGenity and Grant, 1995), a model organism to study cold adaptation in archaea (DasSarma et al., 2013, 2017; Williams et al., 2017), has previously been shown to be genetically accessible (Liao et al., 2016). Several different viruses were shown to infect *Hrr. lacusprofundi* (Nuttall and Dyall-Smith, 1993; Dyall-Smith, 2009d; Krupovič et al., 2010; Atanasova et al., 2012; Porter et al., 2013; Li et al., 2014; Tschitschko et al., 2015; Alarcón-Schumacher et al., 2022; Mercier et al., 2022). Additionally, the formation of plasmid vesicles (PVs), proposed to be an evolutionary precursor of viruses, has only been reported for *Hrr. lacusprofundi* (Erdmann et al., 2017). This makes *Hrr. lacusprofundi* a great model for studying virus-host relationships in archaea, in particular under low temperature, and to investigate the origin and evolution of viruses. A stable genetic system would allow for more in depth studies.

A genetic system, based on an auxotrophic selection marker, that allows in-frame deletions using the pop-in pop-out method has been developed in *H. volcanii* on the basis of the orotate phosphoribosyltransferase gene (*pyrE2*) (Lam and Doolittle, 1992; Wendoloski et al., 2001; Bitan-Banin et al., 2003; Allers et al., 2004; Liao et al., 2021). The Δ*pyrE2* deletion in *H. volcanii* was generated with a non-replicative plasmid containing a non-functional gene construct, created by fusing upstream and downstream flanking regions of the wild-type Δ*pyrE2* gene, and selection with the halobacterial novobiocin resistance gene (*gyrB*) (Bitan-Banin et al., 2003). Pop-in refers to the homologous recombination of plasmid and host chromosome caused by selection pressure applied with novobiocin in the culture media. Excision of the plasmid (pop-out) can either result in the wild-type or the mutated gene remaining in the chromosome; therefore, a fraction of the resulting clones is expected to be mutants with a non-functional target gene. The pop-out can either be initiated by removing the original selection pressure (e.g., novobiocin) or with an additional selection pressure using 5-fluoroorotic acid (5-FOA) and uracil (Bitan-Banin et al., 2003). The practicality of 5-FOA as counter-selectable marker was first described in yeast (Boeke et al., 1984) but has since been widely used for both bacteria and archaea (Liu et al., 2011; Redder and Linder, 2012; Liao et al., 2021; Matsuda et al., 2022; Piatek et al., 2022). In prokaryotes, orotate phosphoribosyltransferase (*pyrE*) and orotidine 5-phosphate decarboxylase (*pyrF*) catalyze the final steps in the pyrimidine biosynthesis pathway. The two enzymes convert 5-FOA to the toxic 5-fluoro-UMP leading to cell death (Redder and Linder, 2012).

However, generating spontaneous *pyrE2* mutants by subjecting the cells to 5-FOA and uracil, has not been successful for *Hrr. lacusprofundi* so far (Liao et al., 2016). Liao et al. (2016) described a method for knocking out the acetamidase *amd3* in *Hrr. lacusprofundi* with a suicide plasmid containing *hmgA* (pJWID1), conferring resistance to pravastatin. The 3-hydroxy-3-methylglutaryl coenzyme A reductase gene (*hmgA*) is involved in the isoprenoid lipid synthesis pathway in archaea. Overexpression of *hmgA* leads to mevinolin, fluvastatin, simvastatin, and pravastatin resistance in haloarchaea (Blaseio and Pfeifer, 1990; Lam and Doolittle, 1992; Wendoloski et al., 2001). However, in practice, pravastatin has proven to be the most reliable selection marker for *Hrr. lacusprofundi* (Liao et al., 2016). Liao et al. (2016) were able to show that the *hmgA* gene was inserted between flanking regions of *amd3*. After double recombination, *hmgA* replaced the target gene on the genome resulting in a successful deletion of the target gene. However, the *hmgA* gene remains in the genome and can interfere with the expression of genes in the same operon. The pravastatin resistance conferred by the permanent insertion into the genome also makes it nearly impossible to use the strain for the knockout of additional genes except when using very high pravastatin concentrations, e.g., 150 μg/mL (Liao et al., 2016). However, consecutive culturing of *Hrr. lacusprofundi* in pravastatin concentrations of 10 μg/mL and low concentrations of mevinolin and simvastatin led to multiple insertions and duplications of the *hmgA* gene into all three chromosomes of the strain (Hamm et al., 2019). Additionally, *Hrr. lacusprofundi* transposases also disrupted genes of the vector used, rendering them non-functional.

The aim of the study was to develop a stable system for the genetic manipulation of *Hrr. lacusprofundi* ACAM34 that uses the pop-in pop-out method and allows for in-frame deletions without leaving the antibiotic resistance gene in the genome. We successfully generated *ΔpyrE2* mutants for two *Hrr. lacusprofundi* ACAM34 strains. Subsequently, we demonstrate that the auxotrophic mutants can be used for in-frame deletions of genes using vectors that have been designed for *H. volcanii*, eliminating the necessity to design entirely new vectors.

## Methods

### Strains and cultivation conditions

Unless otherwise specified, strains that are referred to only by their strain name are all strains of *Halorubrum lacusprofundi ACAM34* (ATCC 49239). *Hrr. lacusprofundi* ACAM34_DSMZ was obtained from the DSMZ (German Collection of Microorganisms and Cell Cultures). *Hrr. lacusprofundi* ACAM34_UNSW is a variant with a reduced genome, derived from an original ACAM34 strain, and has been described in Mercier et al. (2022). The standard medium for cultivation was DBCM2 (Dyall-Smith, 2009b), with slight modifications. Standard DBCM2 medium contained 180 g/L NaCl, 25 g/L MgCl, 29 g/L MgSO_4_·7H_2_O, 5.8 g/L KCl (all Merck, Germany) as well as 0.3 g/L peptone and 0.05 g/L yeast extract (both by Oxoid, Thermo Fisher Scientific, United States), dissolved in ddH_2_O, and adjusted to pH 7.5 with 1 M Tris–HCl pH 8.0 prior to autoclaving. After autoclaving, supplements were added 6 mL/L 1 M CaCl_2_, 2 mL/L K_2_HPO_4_ buffer (Dyall-Smith, 2009b), 4.4 mL/L 25% sodium pyruvate, 5 mL/L 1 M NH_4_Cl, 1 mL/L SL10 trace elements solution (Dyall-Smith, 2009c), and 3 mL/L vitamin 10 solution (Dyall-Smith, 2009c). Initially, agar plates were prepared with ddH_2_O and 18 g/L Oxoid™ agar. However, tap water was found to be best suited to support growth of ACAM34 on agar plates. Starting with the plating of pTA131_*hmgA*_Δ*trpA* transformants from liquid onto agar plates, the components for agar plates were dissolved in tap water and supplemented with 18 g/L Difco™ agar (BD, United States). We are aware that tap water is prone to contaminations and changes in composition, and indeed, we observed slight growth of auxotrophic mutants on selection plates in some seasons of the year. However, using tap water allowed for growth of ACAM34 colonies on plates in a reasonable time, while ddH_2_O often did not result in colonies. For the rich medium DBCM2+, peptone and yeast extract concentrations were adjusted to 1 g/L and 0.5 g/L, respectively. Medium DBCM2 contained 5 g/L casamino acids (Oxoid) instead. For the generation of ACAM34_UNSWΔ*pyrE2*, the strain was plated on Hv-Cab solid agar as described in de Silva et al. (2021). Liquid cultures were inoculated at 28°C, because biofilm formation was often observed at 37°C (published optimal growth temperature) in liquid; however, plates were incubated at 37°C leading to a timely (7–10 days) formation of colonies. For light microscopy images, cells in mid-exponential growth were fixed for 1 h at room temperature (RT) with 1% glutaraldehyde, immobilized on 4% agar slides, and imaged on a Zeiss AxioPhot microscope with AxioCam MRm.

*Escherichia coli* strains (DH5alpha and C2925) were grown at 37°C, either in liquid lysogeny broth (LB) medium (10 g/L tryptone, 5 g/L yeast extract, 10 g/L NaCl_2_) (AppliChem, Germany and by Merck) in ddH_2_O at pH 7.0 or on LB plates with 15 g/L Bacto™ agar (BD). Ampicillin was used as a selection agent at a final concentration of 100 μg/mL.

### Molecular cloning procedures

DNA extractions were performed with the Bioline Isolate II Genomic DNA Kit (Meridian Bioscience, Cincinnati, United States). Plasmid extractions were performed using the Bioline Isolate II Plasmid Mini Kit, and DNA purification (from PCR or gel electrophoresis) was performed using the Bioline Isolate II PCR and Gel Clean-Up Kit. All extractions were performed following the manufacturer’s instructions. Plasmids used in this study are summarized in Supplementary Table 1. The primers are listed with their respective annealing temperatures and targets in Supplementary Table 2. Amplification of archaeal genes was performed with Q5 Polymerase (NEB) as follows: Approximately 100 ng of template DNA was added to PCR master mix (per 100 μL; 1x reaction buffer (NEB, Ipswich, United States), 1x high GC enhancer (NEB), 40 μM dNTPS (NEB), 100 μM of forward and reverse primer each (biomers.net, Ulm, Germany), and 1 μL Q5 polymerase (NEB) and PCR was performed with the following program: Initial denaturation at 96°C for 1 min, 30x cycles at denaturation 96°C for 30 s, annealing (temperature in Supplementary Table 2) for 30 s, elongation at 72°C (1 min/kb), a final elongation at 72°C for 10 min, and storage at 12°C. When generating PCR fragments for cloning (with restriction sides included), a two-step PCR was performed with 10 cycles using the annealing temperature for the part of the primer that binds the template and 30 cycles with the annealing temperature calculated for the entire length of the primer, including additional bases (e.g., the restriction side). Overlapping PCRs were performed with Phusion Polymerase (NEB) according to the manufacturer’s instructions with 5% DMSO in the reaction in two steps: 24 cycles without primers and 35 cycles with fresh dNTPs, Phusion Polymerase, and the outermost primers added to the PCR mix (forward primer used to generate the upstream fragment and reverse primer used to generate the downstream fragment). Restriction digest was performed in 30 μl reactions with 1 μL enzyme (as specified for individual cloning reactions), 3 μL 10x reaction buffer, and 1–3 μg of DNA for 2 h at 37°C with an inactivation step of 20 min at 70°C. Ligation reactions were performed in 20 μl volume with 1 μL T4 Ligase (NEB), 2 μL 10x reaction buffer approximately 100 ng of vector and 300 ng of insert for 2 h at RT.

Ligated constructs were transformed into *E. coli* DH5alpha, *via* heat-shock for 1 min at 42°C and plated on LB medium agar plates amended with 100 μg/mL ampicillin, followed by transformation into *E. coli* C2925 (NEB) for demethylation (Liao et al., 2021). Both *E. coli* and *Hrr. lacusprofundi* colonies were screened *via* colony PCR after transformation. Scratched cell material from each colony was spread on a fresh agar plate and the remaining cell material on the sterile pipet tip was either directly swirled in Q5 PCR master mix aliquots (for *E. coli*) or in 10 μL of sterile ddH_2_O (for *Hrr. lacusprofundi*), from which 1 μL was used per colony as the PCR template as described above. Plasmid constructs were verified by Sanger sequencing in-house on the ABI 3130xl Sequence Analyzer (Applied Biosystems, Foster City, United States).

### Plasmid construction

The PCR construct to attempt knockout of the *pyrE2* gene by homologous recombination in *Hrr. lacusprofundi* was generated by PCR amplification of the upstream fragment using Pyr_USF and Pyr_USR, the downstream fragment using Pyr_DSF and Pyr_DSR and subsequent fusion of the two fragments by overlapping PCR (as described previously, e.g., Dattani et al., 2022; Supplementary Table 2). The PCR construct was either used directly in a transformation or cloned into the pTA131 vector (Allers et al., 2004) using *Hind*III and *BamH*I restriction sites. For pTA131_*ΔpyrE2*_*hmgA*, the *hmgA* gene was amplified from pJWID1 (Wendoloski et al., 2001; Liao et al., 2016) using prav_F and prav_R in a two-step PCR. The resulting product was then cloned into pTA131 using *Xba*I and *Not*I restriction sites.

For pTA131_*hmgA*_Δ*trpA*, the *hmgA* gene was cloned into pTA131 as described for pTA131_*ΔpyrE2*_*hmgA.* Subsequently, upstream (1273UF and 1273UR) and downstream (1273DF and 1273DR) flanking regions of the *trpA* gene Hlac_1273 were amplified separately and fused together in consecutive PCR reactions (as described above). The primers included restriction sites for *Hind*III and *BamH*I, through which the PCR product was cloned into the pTA131_*hmgA* vector. The pTA131_*hmgA*_*ΔHlac_2746* and pTA132_*ΔHlac_2746* vectors were constructed the same way, *via* amplification and fusion of flanking regions of the putative GTPase gene Hlac_2746 (Hlac_2746UF, Hlac_2746UR, Hlac_2746DF, and Hlac_2746DR), followed by insertion into the plasmids (see Supplementary Table 1) with *HindIII* and *BamHI* as described above.

### Genetic manipulation of *Hrr. lacusprofundi*

All plasmids were transformed into *E. coli* C2925 (NEB) for demethylation before transformation into *Hrr. lacusprofundi* strains. Transformation of *Hrr. lacusprofundi* was performed as described earlier (Liao et al., 2016) following a PEG_600_-based protocol with slight modifications. The incubation of the cells with DNA after the addition of PEG_600_ lasted 70 min. Incubation in regeneration buffer was performed overnight at 30°C. For the transformation with pTA132_*ΔHlac_2746*, the *Hrr. lacusprofundi* ACAM34_UNSWΔ*pyrE2*Δ*trpA* transformants were resuspended in regeneration solution containing 10x casamino acids replacing 10x YPC with trace elements and vitamins added in the ratio common for DBCM2 medium (Dyall-Smith, 2009a). Transformants were plated on selective media and liquid cultures were inoculated as well. For *pyrE2* deletions, both ACAM34_UNSW and ACAM34_DSMZ were directly plated on plates containing 150 μg/mL 5-fluoroorotic acid (5-FOA) and 50 μg/mL uracil. For *trpA* deletions, ACAM34_UNSW and ACAM34_UNSWΔ*pyrE2* were grown in DBCM2- media with pravastatin at concentrations indicated. Pop-out was performed on plates containing 150 μg/mL 5-FOA and 50 μg/mL uracil. ACAM34_UNSWΔ*pyrE2*Δ*trpA* mutants S1 and S2 were plated on DBCM2-media with uracil (50 μg/mL) selecting for pTA132_*ΔHlac_2746* which includes a functional *trpA* gene. The deletion mutant of *Hlac_2746* in ACAM34_UNSWΔ*pyrE2* was subsequently generated by transformation with the pTA131_*hmgA*_ *ΔHlac_2746* plasmid and a two-step selection as described above for the *trpA* deletion mutants. We screened liquid cultures and single colonies for the correct insert by colony PCRs and Sanger sequencing as described above.

### Genome sequencing and analysis of *trpA* mutants

Library preparation (FS DNA Library, NEBNext^®^ Ultra™) and sequencing (Illumina HiSeq3000, 2 × 150 bp, 1 Gigabase per sample) was performed at the Max Planck-Genome-Centre Cologne (Cologne, Germany). For genome analysis, reads were mapped to ACAM34_UNSW genome using “geneious mapper” (Geneious Prime^®^ 2022.2.1) with medium-low sensitivity and default settings. No reads could be recruited for the described gene deletion.

## Results

### Attempts to generate uracil auxotroph mutants by directed double crossover resulted in spontaneous mutants with unexpected deletions

**Figure 1:**
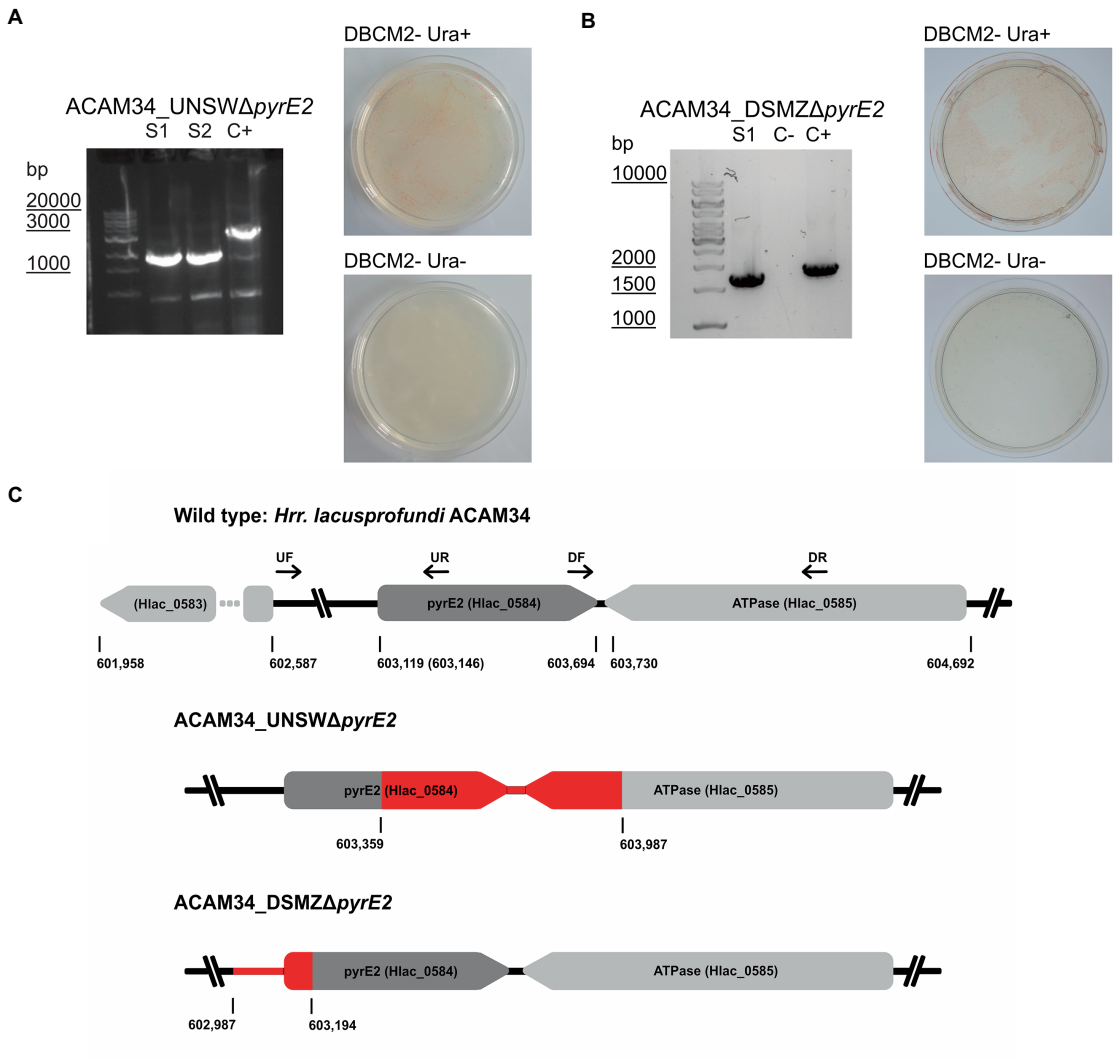
Successful *pyrE2* knockout in *Hrr. lacusprofundi* ACAM34 strains. **(A)** Characterization of ACAM34_UNSWΔ*pyrE2* mutants. Left: PCR on the *pyrE2* locus of the two knockout strains S1 and S2 (see also Supplementary Figure 2) and a wild-type ACAM34_UNSW control (C+). Right: Growth of ACAM34_UNSWΔ*pyrE2* on DBCM2- supplemented with uracil (top), and growth defect on DBCM2− without uracil (bottom). **(B)** Characterization of ACAM34_DSMZΔ*pyrE2*. Left: PCR on the *pyrE2* locus of the knockout mutant, ACAM34_DSMZ wild-type DNA served as the positive control (+C) (see also Supplementary Figure 5). Right: Growth of ACAM34_DSMZΔ*pyrE2* on DBCM2- supplemented with uracil (top), and growth defect on DBCM2- without uracil (bottom). **(C)** Schematic representation of the genomes including the wild-type strain ACAM34_UNSW with the position (bp) of *pyrE2* and neighboring genes. ACAM34_DSMZ and ACAM34_UNSW deviate in the length of the annotated *pyrE2* gene, the different position is marked in brackets for ACAM34_DSMZ. The primer binding sites for upstream (UF, UR) and downstream (DF, DR) flanking regions are represented as arrows. The deletion in the ACAM34_UNSWΔ*pyrE2* S1 and S2 clones is identical and highlighted in red. The deletion in ACAM34_DSMZΔ*pyrE2* is also highlighted in red.

Orotate *phosphoribosyltransferase* (*pyrE2)* is an ideal target to generate auxotrophic strains for genetic manipulation. Previous attempts to generate spontaneous *ΔpyrE2* mutants of *Hrr. lacusprofundi* with uracil (U) and 5-fluoroorotic acid (5-FOA) treatments (Bitan-Banin et al., 2003) have been unsuccessful (Liao et al., 2016). Therefore, we attempted to generate *pyrE2* deletions by transforming *Hrr. lacusprofundi* strain ACAM34_UNSW with a knockout (KO) PCR product composed of the fused up- and downstream flanks of the *pyrE2* gene of ACAM34, followed by selection on U/5-FOA plates (Figure 1C). In a successful double-crossover homologous recombination, the construct would be inserted into the genome and mutation of wild-type (WT) genes would be induced by 5-FOA. However, none of the obtained cultures tested showed the expected deletion in the *pyrE2* gene (Supplementary Figure 1), even though single-nucleotide polymorphisms (SNPs) and small insertions or deletions cannot be ruled out. Next, we cloned the KO PCR product into pTA131, a non-replicating vector designed for *H. volcanii* (Allers et al., 2004). We then transformed pTA131_Δ*pyrE2* and pTA131 as control into *Hrr. lacusprofundi* ACAM34_UNSW and ACAM34_DSMZ followed by selection on U/5-FOA plates. Similar growth was detected for both strains, and for both the control and pTA131_Δ*pyrE2*. The majority of colonies tested for ACAM34_UNSW revealed either wild-type size of the gene (approximately 60%) or an increased size of the gene, indicating the insertion of a transposase (approximately 40%) (Supplementary Figure 2) that was confirmed for one representative clone by sequencing. One out of 64 tested colonies revealed a PCR product indicative of a gene deletion; however, wild-type gene copies could still be detected. Haloarchaea are known for being polyploid (Maurer et al., 2018; Ludt and Soppa, 2019) and the mixture of wild-type gene and mutant gene is likely the result of the heterozygous genome of this particular clone. After re-streaking, we obtained a clone (ACAM34_UNSWΔ*pyrE2* S1) with a clean PCR signal for a gene deletion without any apparent background levels of wild-type genes present (Figure 1A; Supplementary Figure 2B). The strain was unable to grow in absence of uracil, confirming the inactivation of the *pyrE2* gene. Surprisingly, sequencing of the genomic region revealed a deletion of 628 base pairs (bp) including the C-terminus of *pyrE2* [amino acids (aa) 73–191] and the C-terminus (last 84 aa) of a downstream gene, Hlac_0585, an ATPase associated with various cellular activities encoded on the opposite strand (Figure 1C). We conclude that this deletion is a spontaneous deletion, because it does not reflect the region we targeted with pTA131_Δ*pyrE2*.

**Figure 2:**
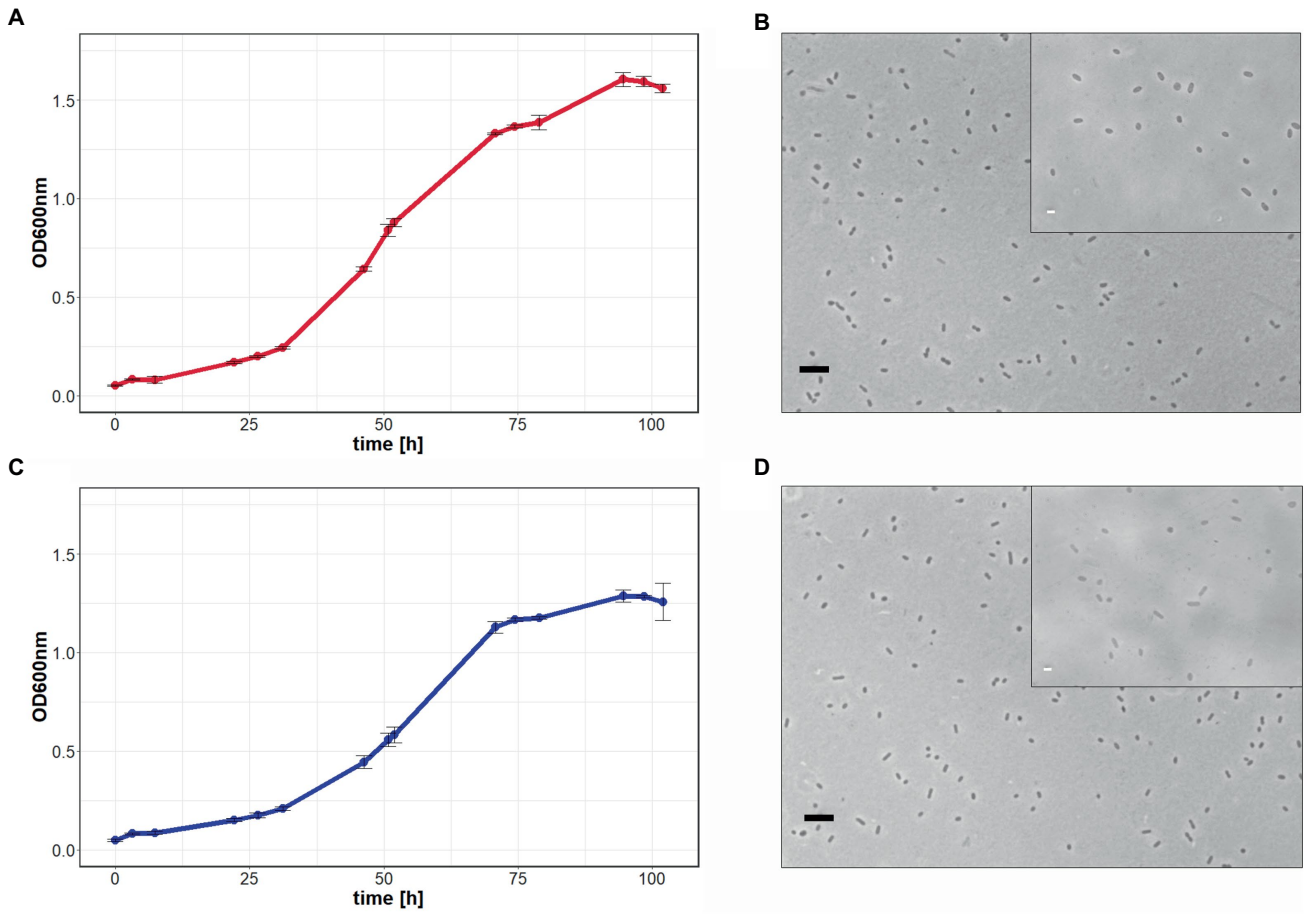
Growth comparison of *Hrr. lacusprofundi* ACAM34_UNSW and ACAM34_UNSWΔ*pyrE2.* The growth of wild-type **(A)** and mutant cells **(C)** in rich medium was observed by optical density measurements at 600 nm (OD_600 nm_) over 102 h. Points represent the average values and each time point measured, including the standard deviation represented by error bars (*n* = 3). Light microscopy images of wild-type **(B)** and mutant cells **(D)** represent one of the three biological replicates (Supplementary Figure 4). Larger image at 400× magnification, black scale bar indicates 10 μm, inlet at 1,000× magnification, white scale bar indicates 2 μm.

In a third attempt to obtain a clean *pyrE2* deletion in ACAM34_UNSW, we cloned the *hmgA* gene from pJWID1, conferring resistance against pravastatin (Liao et al., 2016), into pTA131_*ΔpyrE2*. Pravastatin selection was used to force the insertion of the KO construct into the genome (pop-in) and then U/5-FOA to force the vector to excise again (pop-out). The construct was transformed into ACAM34_UNSW and two colonies that were positive in PCR screens for the *hmgA* gene were chosen for pop-out after two successive dilutions in 2.5 μg/mL pravastatin. As with previous attempts, a double-crossover homologous recombination could not be achieved (Supplementary Figure 3). Out of 32 screened colonies one clone with a deletion was identified (Supplementary Figure 2C), hereafter referred to as ACAM34_UNSWΔ*pyrE2*_S2 (Figure 1A). Sequencing of the genomic region also showed a deletion identical to ACAM34_UNSWΔ*pyrE2*_S1 that we conclude to be a spontaneous mutation. We are aware that this mutation also affects a second gene. However, we did not detect any major defects in neither growth (Figure 2C) nor morphology (Figure 2D and Supplementary Figure 4). The mutants are furthermore unable to grow on medium without uracil (Figure 1A). Since an in-frame deletion could not be achieved, we selected this auxotrophic mutant for further studies.

A similar phenomenon occurred in ACAM34_DSMZ. None of 40 tested colonies from the transformation with pTA131_*ΔpyrE2*, showed a change in size of the target region. However, out of eight colonies screened from a control transformation with water, two showed increased size and one a reduced size (ACAM34_DSMZΔ*pyrE2*) (Figure 1B; Supplementary Figure 5). Sequencing of the PCR product revealed a short deletion that expands over the promotor region of the gene and the first 16 aa (Figure 1C). After re-streaking of the strain, we were able to isolate a colony that had a clean PCR result for the gene deletion and was not able to grow without uracil (Figure 1B).

### Successful in-frame deletion of the *trpA* gene in uracil auxotrophic strains required a double selection

To test whether the newly generated auxotrophic *pyrE2* mutant ACAM34_UNSWΔ*pyrE2*, is stable and suitable to be used for genetic manipulation, we targeted the alpha subunit of the tryptophan synthase (*trpA*, Hlac_1273) that is also commonly used in *H. volcanii* as a second auxotrophic marker (Allers et al., 2004).

**Figure 3:**
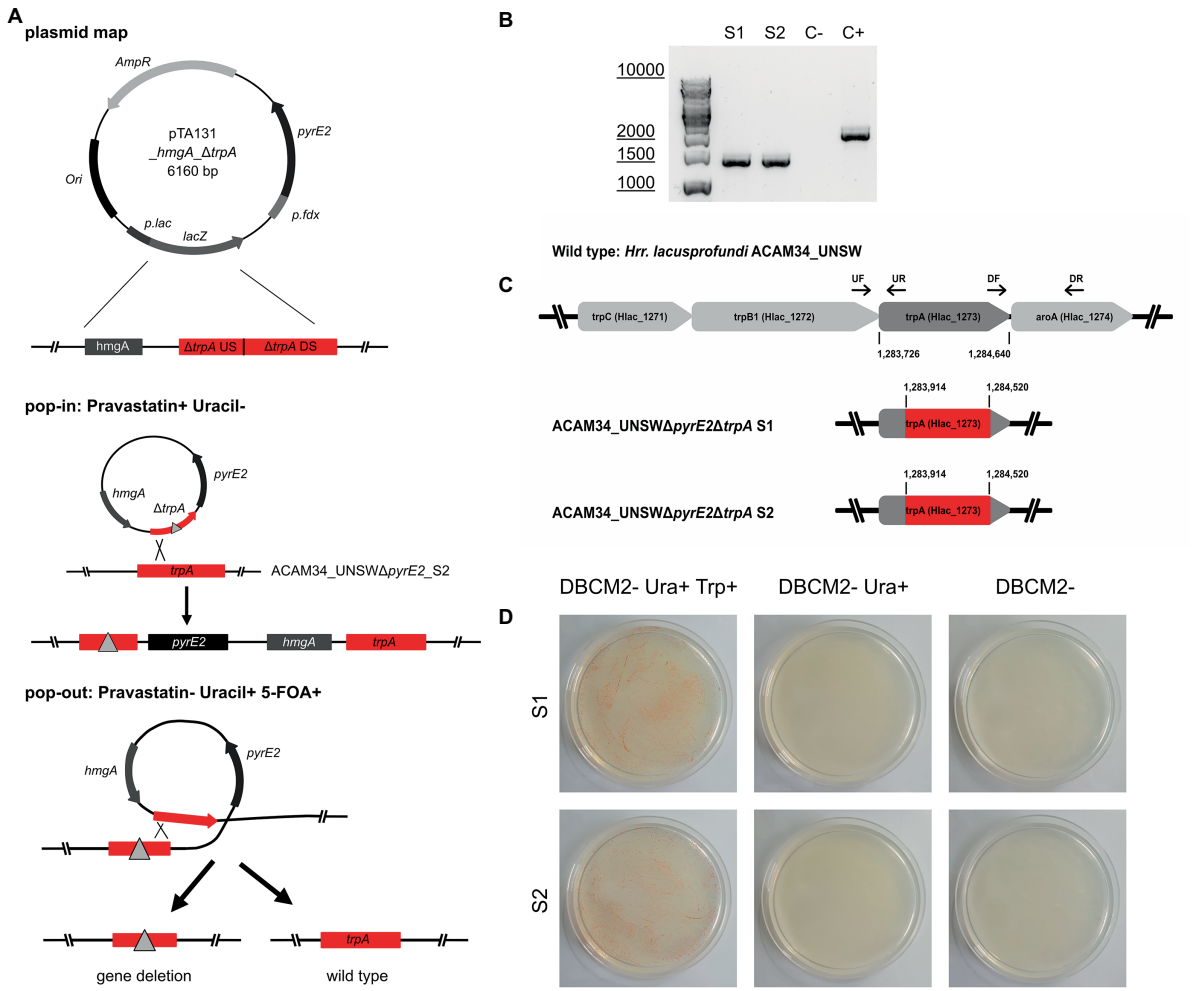
Successful *trpA* knockout in *Hrr. lacusprofundi* ACAM34_UNSWΔ*pyrE2*. **(A)** Recombination strategy used to generate ACAM34_UNSWΔ*pyrE2* Δ*trpA via* double selection. A vector based on pTA131 (Allers et al., 2004) (with *pyrE2* for uracil synthesis), was used to insert a pravastatin resistance gene (*hmgA*), as well as a non-functional copy of the *trpA* gene created by the fusion of upstream (US) and downstream (DS) fragments of the gene. Both the non-functional gene (represented by a triangle) and the functional wild-type *trpA* gene are marked in red. Crossover at the *trpA* genes leads to the integration of the plasmid into the genome (pop-in) using uracil depletion and the addition of pravastatin as selection pressure. Excision of the plasmid (pop-out) is enforced by the addition of 5-FOA and uracil, through which either the non-functional construct (left) or the wild-type gene (right) remains in the genome. **(B)** PCR on the *trpA* locus of the two knockout strains S1 and S2, with a wild-type ACAM34_UNSW control (C+) and a negative control (C−). **(C)** Operon encoding genes for tryptophan synthesis in the wild-type strain ACAM34_UNSW including the position (bp) of *trpA* and the primer binding sites for upstream (UF, UR) and downstream (DF, DR) flanking regions amplified for the deletion construct. Deletion in ACAM34_UNSWΔ*pyrE2*Δ*trpA* S1 and in ACAM34_UNSWΔ*pyrE2*Δ*trpA* S2 highlighted in red. **(D)** Growth of ACAM34_UNSWΔ*pyrE2*Δ*trpA* S1 (top) and S2 (bottom) on DBCM2 supplemented with uracil and tryptophan (left), growth defect on DBCM2 with uracil but without tryptophan (middle), growth defect on DBCM2 (right).

Both ACAM34_UNSW and ACAM34_UNSWΔ*pyrE2*_S2 were transformed with pTA131_ *hmgA*_Δ*trpA* containing a non-functional *trpA* construct (Figure 3A). While selection of ACAM34_UNSW transformants was only based on pravastatin resistance (2.5 μg/mL) conferred by *hmgA*, ACAM34_UNSWΔ*pyrE2* transformants were selected for the presence of the plasmid with both pravastatin (*hmgA*) and uracil depletion, forcing the uptake of the plasmid with the *H. volcanii pyrE2* gene (83% amino acid identity to ACAM34_UNSW). Growth of transformants was only achieved in liquid cultures and not on solid agar plates. However, while both ACAM34_UNSW and ACAM34_UNSWΔ*pyrE2* were growing in selective media, the presence of the plasmid could only be detected in ACAM34_UNSWΔ*pyrE2* (Supplementary Figure 6). Since ACAM34_UNSW controls transformed with an empty vector (pTA131) or water also showed growth in liquid, we conclude that 2.5 μg/mL pravastatin was not sufficient for selection (Supplementary Figure 6). The slight growth of ACAM34_UNSWΔ*pyrE2* in liquid without uracil was only observed shortly after transformation and we assume that contaminations with uracil in the regeneration buffer could have supported the growth. The ACAM34_UNSWΔ*pyrE2* culture transformed with pTA131_*hmgA*_Δ*trpA* was plated on selective media (2.5 μg/mL pravastatin, DBCM2-) and single colonies were screened by PCR. While the presence of the *hmgA* gene was detectable in all clones, PCR with primers targeting the *trpA* gene on the genome revealed mixed signal of both WT and mutated *trpA* genes in all samples (Supplementary Figure 7). We concluded that, similar to other haloarchaea (Maurer et al., 2018), *Hrr. lacusprofundi* exhibits several copies of its genome within one cell. The PCR signals from the targeted genomic region suggested that some chromosome copies had already excluded the plasmid, resulting in either a mutated or a wild-type *trpA*, and some wild-type copies might have not taken up the plasmid. To force the cell to exclude chromosome copies without the inserted plasmid before pop-out, we selected three colonies for culturing in increased (7.5 μg/mL) pravastatin concentrations in liquid culture; however, after four generations (G4), no changes were detected by PCR. The following generations were grown in increasing pravastatin concentrations of 10, 15, 20, and 40 μg/mL (G8) and both G4 and G8 were selected for pop-out on U/5-FOA plates. Colonies of G4 and G8 on pop-out plates were screened with genomic primers for the *trpA* gene. G4 colonies, with a PCR signal, all showed the wild-type gene with one exception that resulted in a mixture of wild-type and mutant *trpA* (Supplementary Figure 8). The majority of G8 pop-out clones showed a mixture of wild-type and mutant *trpA*, and five clones with a clean *trpA* deletion were identified (Supplementary Figure 9). These five clones were transferred into both full media (containing tryptophan and uracil) and selective media (with uracil only) to test for tryptophan auxotrophy. Four clones reverted back to WT and were able to grow in selective media, but one clone referred to as ACAM34_UNSWΔ*pyrE2*Δ*trpA* was unable to grow without tryptophan (Figure 3D) and PCR confirmed a corrupted *trpA* gene (Figure 3B; Supplementary Figure 10).

After replating, we selected two clones ACAM34_UNSWΔ*pyrE2*Δ*trpA* (S1 and S2) for genome sequencing. For both ACAM34_UNSWΔ*pyrE2*Δ*trpA* S1 and S2, no reads could be detected for the target region, between the upstream and downstream flanking regions of the non-functional *trpA* construct in pTA131_*hmgA*_Δ*trpA* (Figure 3C). The gap extends from aa63 to aa265, creating a clean 202aa deletion in both deletion strains without affecting any other genes or non-coding regions (Figure 3C).

### A vector designed for *trpA* selection in *H. volcanii* can be used for selection in the tryptophan auxotrophic mutant ACAM34_UNSWΔ*pyrE2*Δ*trpA*

All the plasmids created in this study were derived from plasmids originally created for the haloarchaeal model organism *H. volcanii*. For convenience in future experiments, we decided to test whether other plasmids designed for *H. volcanii* could be effectively used in *Hrr. lacusprofundi* without modifications. For this purpose, we selected a putative SAR1 family GTPase (Hlac_2746, Erdmann et al., 2017) as a target gene for a gene knockout. We chose pTA132 (Allers et al., 2004), a non-replicating vector including a functional *H. volcanii trpA* gene. In this case, plasmid uptake would be solely dependent on the *trpA* selection, allowing us to assess the efficiency of *trpA* selection in the newly generated ACAM34_UNSWΔ*pyrE2*Δ*trpA* strain.

We transformed both ACAM34_UNSWΔ*pyrE2*Δ*trpA* strains (S1 and S2) with pTA132_ Δ*Hlac_2746* containing a non-functional Hlac_2746 construct, followed by selection with media lacking tryptophan [DBCM2- and uracil (50 μg/mL)]. Growth was only detected in liquid cultures of S2 transformants that went through regeneration in 10x casamino acids derived medium instead of 10x YPC as in previous transformations (see Methods). No growth was detected for transformants of S1 neither on plates nor in liquid culture.

Without an additional antibiotic selection with pravastatin, we expected a lower selection efficiency and cultivated the transformants over successive generations before pop-out in selective media. After four generations (G4) we screened 34 colonies for plasmid insertion with primers binding in the region outside of Hlac_2746 and a long elongation time (6 min) to detect inserted plasmids (Supplementary Figure 11). Most colonies showed a mixture of both wild-type gene and inserted plasmid (approximately 95%), including two liquid cultures that represented the fifth generation (G5). We concluded that several copies of the genome are present in these cells some with an insertion and some without, as we observed earlier. We conclude that pTA132 designed for *trpA*-mediated selection in *H. volcanii* was also effective for *trpA*-mediated selection in *Hrr. lacusprofundi*. However, *trpA* selection alone does not seem to be sufficient to force the cells to include the plasmid into all chromosome copies.

We plated G5 on rich medium (DBCM2+ 50 μg/mL uracil and tryptophan) to allow pop-out. After three successive passages (G6-G8) in rich medium, colonies (*n* = 22) screened in G9 either still retained the plasmid (41%), showed a mixture of wild-type Hlac_2746 and mutated Hlac_2746 (18%), a mixture of plasmid insertion and wild type (9%), a mixture of plasmid insertion, wild-type and mutated Hlac_2746 (23%), or no signal at all (9%) (Supplementary Figure 12). Since the insertion of the plasmid into all chromosome copies was already inefficient, it is not surprising that we could not identify any deletion mutants without background signal.

Subsequently, we attempted the knockout of *Hlac_2746* in ACAM34_UNSWΔ*pyrE2* by double selection, using uracil depletion and pravastatin selection as described above. After transformation with pTA131_*hmgA*_Δ*Hlac_2746*, colonies that had successfully taken up the plasmid were subjected to increasing pravastatin concentrations over 8 generations in liquid culture (DBCM2-) followed by pop-out on U/5-FOA plates. Out of 62 colonies, 6 showed a PCR product smaller than wild-type *Hlac_2746* (Supplementary Figure 13), and three of them were confirmed to contain the target deletion by sequencing.

## Discussion

The previously reported genetic system for *Hrr. lacusprofundi* that is based on antibiotic selection, inserting the *hmgA* gene into the target gene (Liao et al., 2016), does not exclude effects on gene expression downstream of the deletion. Additionally, it was shown later that *hmgA* can be incorporated from a plasmid into the genome of *Hrr. lacusprofundi*, making it a volatile selection marker (Hamm et al., 2019). In this study, we aimed to generate a uracil auxotrophic mutant for robust selection and for the development of a genetic system allowing us to perform in-frame deletions.

Since generation of spontaneous *pyrE2* mutants on U/5-FOA plates was unsuccessful in a previous study (Liao et al., 2016), we attempted to generate *pyrE2* mutants by double crossover. All three attempts, using either a pure PCR product of the deletion construct or a vector including the deletion construct, with and without antibiotic selection, failed to produce the target deletion. However, we obtained spontaneous *pyrE2* mutants from these experiments for two strains of *Hrr. lacusprofundi* (ACAM34_UNSW and ACAM34_DSMZ). Two auxotrophic mutants of ACAM34_UNSW experienced the exact same deletion in two independent experiments (Figure 1B), suggesting that this deletion, including the 5′-part of a downstream gene, is not random. The strain does not show significant growth defects (Figure 2); therefore, ACAM34_UNSWΔ*pyrE2* was chosen as suitable candidate for further genetic manipulation.

To attempt the generation of an in-frame gene deletion, we chose the *trpA* gene as a target. We tested both wild-type ACAM34_UNSW and the uracil auxotroph ACAM34_UNSWΔ*pyrE2*. Antibiotics selection alone was not sufficient to achieve the uptake of the plasmid in ACAM34_UNSW wild type. However, double selection of the auxotrophic mutant with antibiotic and uracil depletion in ACAM34_UNSWΔ*pyrE2* resulted in a successful uptake. A clean deletion mutant was only isolated from an ACAM34_UNSWΔ*pyrE2* transformant that was subjected to increasing concentrations of pravastatin (up to 40 μg/mL) before pop-out.

We subsequently tested the newly generated ACAM34_UNSWΔ*pyrE2*Δ*trpA* auxotrophic mutant for the efficiency of *trpA-*mediated selection without the additional selection markers *hmgA* and *pyrE2*. The plasmid, including a functional *trpA* gene derived from *H. volcanii*, was successfully taken up under tryptophan depleted conditions. However, an insertion into all chromosome copies could not be achieved without additional selection. Subsequently, we were not able to obtain clean deletion mutants. Haloarchaea are known for polyploidy (Maurer et al., 2018; Ludt and Soppa, 2019). Therefore, successive cultivation under selective conditions is essential to remove any remaining wild-type copies of the gene and selection for auxotrophic markers alone was not efficient in *Hrr. lacusprofundi* over four generations. Extending the selection over more generations might be sufficient, however, is also very time consuming. Alternatively, phosphate starvation, to reduce the number of genome copies (Maurer et al., 2018), could be used to add additional selection pressure. Nevertheless, the double selection with pravastatin and U/5-FOA yielded several clean deletion mutants that were not obtained with *trpA-*mediated selection when targeting the same gene. Both the selection with antibiotics alone and the auxotrophic marker alone proved to be unsuccessful. We conclude that the combination of an auxotrophic selection marker together with antibiotics selection is the most time efficient strategy to achieve clean deletion mutants in *Hrr. lacusprofundi*. Indeed, we and other collaborating laboratories have successfully generated several knockout mutants in *Hrr. lacusprofundi* ACAM34_UNSW using this strategy (unpublished results).

We successfully used plasmids originally designed for the haloarchaeal model organism *H. volcanii* for the genetic manipulation of *Hrr. lacusprofundi*. This opens up the possibility to use the entire genetic tool box available for *H. volcanii* (Dattani et al., 2022; Harrison and Allers, 2022) in *Hrr. lacusprofundi*. In addition to derivatives of the pTA131/132 used for gene knockouts (Liao et al., 2021), vectors for protein overexpression (Allers, 2010), recombinant luciferase (Davis et al., 2020), and fluorescent proteins (Reuter and Maupin-Furlow, 2004) are available to visually track gene expression (Duggin et al., 2015), protein interactions (Winter et al., 2018), and protein location inside of cells (Lestini et al., 2015; Liao et al., 2021; Nußbaum et al., 2021).

In conclusion, we successfully generated uracil and tryptophan auxotrophic mutants of *Hrr. lacusprofundi* ACAM34 and demonstrate that uracil auxotrophic mutants can be genetically manipulated using well-established plasmids designed for the model organism *H. volcanii*.

## Data availability statement

The datasets presented in this study can be found in online repositories. Raw data from genome sequencing are available in the NCBI repository (https://www.ncbi.nlm.nih.gov) under BioProjectID: PRJNA894865.

## Author contributions

LJG performed the majority of the experimental work. SE performed some of the experiments, conceived, and led the study. LJG and SE performed the primary writing of the manuscript. ID led the initial phase of the study. All authors participated in the analysis and interpretation of the data and contributed to the writing of the manuscript.

## Funding

Funding was provided by the Volkswagen Foundation (reference 98 190), Germany, and the Max Planck Society (Munich, Germany). Part of this work was supported by the Australian Research Council Future fellowship to ID (FT160100010).

## Conflict of interest

The authors declare that the research was conducted in the absence of any commercial or financial relationships that could be construed as a potential conflict of interest.

## Publisher’s note

All claims expressed in this article are solely those of the authors and do not necessarily represent those of their affiliated organizations, or those of the publisher, the editors and the reviewers. Any product that may be evaluated in this article, or claim that may be made by its manufacturer, is not guaranteed or endorsed by the publisher.

## References

[ref1] Aguirre Sourrouille Z. Schwarzer S. Lequime S. Oksanen H. M. Quax T. E. F. (2022). The viral susceptibility of the *Haloferax* species. Viruses 14:1344. doi: 10.3390/v14061344, PMID: 35746816 PMC9229481

[ref2] Alarcón-Schumacher T. Naor A. Gophna U. Erdmann S. (2022). Isolation of a virus causing a chronic infection in the archaeal model organism *Haloferax volcanii* reveals antiviral activities of a provirus. Proc. Natl. Acad. Sci. U. S. A. 119:e2205037119. doi: 10.1073/pnas.2205037119, PMID: 35994644 PMC9436352

[ref3] Allers T. (2010). Overexpression and purification of halophilic proteins in *Haloferax volcanii*. Bioeng. Bugs 1, 290–292. doi: 10.4161/bbug.1.4.11794, PMID: 21327063 PMC3026470

[ref4] Allers T. Ngo H.-P. Mevarech M. Lloyd R. G. (2004). Development of additional selectable markers for the halophilic archaeon *Haloferax volcanii* based on the *leuB* and *trpA* genes. Appl. Environ. Microbiol. 70, 943–953. doi: 10.1128/aem.70.2.943-953.2004, PMID: 14766575 PMC348920

[ref5] Atanasova N. S. Demina T. A. Buivydas A. Bamford D. H. Oksanen H. M. (2015). Archaeal viruses multiply: Temporal screening in a solar saltern. Viruses 7, 1902–1926. doi: 10.3390/v7041902, PMID: 25866903 PMC4411682

[ref6] Atanasova N. S. Roine E. Oren A. Bamford D. H. Oksanen H. M. (2012). Global network of specific virus–host interactions in hypersaline environments. Environ. Microbiol. 14, 426–440. doi: 10.1111/j.1462-2920.2011.02603.x, PMID: 22003883

[ref7] Bitan-Banin G. Ortenberg R. Mevarech M. (2003). Development of a gene knockout system for the halophilic archaeon *Haloferax volcanii* by use of the *pyrE* gene. J. Bacteriol. 185, 772–778. doi: 10.1128/jb.185.3.772-778.2003, PMID: 12533452 PMC142808

[ref8] Blaseio U. Pfeifer F. (1990). Transformation of *Halobacterium halobium*: Development of vectors and investigation of gas vesicle synthesis. Proc. Natl. Acad. Sci. U. S. A. 87, 6772–6776. doi: 10.1073/pnas.87.17.6772, PMID: 11607099 PMC54619

[ref9] Boeke J. D. LaCroute F. Fink G. R. (1984). A positive selection for mutants lacking orotidine-5′-phosphate decarboxylase activity in yeast: 5-fluoro-orotic acid resistance. Mol. Gen. Genet. 197, 345–346. doi: 10.1007/bf00330984, PMID: 6394957

[ref10] Correa T. Abreu F. (2020). “Chapter 20 – Antarctic microorganisms as sources of biotechnological products” in Physiological and biotechnological aspects of extremophiles. eds. Salwan R. Sharma V. (Cambridge: Academic Press), 269–284.

[ref11] DasSarma S. Capes M. D. Karan R. DasSarma P. (2013). Amino acid substitutions in cold-adapted proteins from *Halorubrum lacusprofundi*, an extremely halophilic microbe from Antarctica. PLoS One 8:e58587. doi: 10.1371/journal.pone.0058587, PMID: 23536799 PMC3594186

[ref12] DasSarma P. Laye V. J. Harvey J. Reid C. Shultz J. Yarborough A.. (2017). Survival of halophilic archaea in Earth’s cold stratosphere. Int. J. Astrobiol. 16, 321–327. doi: 10.1017/S1473550416000410

[ref13] Dattani A. Harrison C. Allers T. (2022). “Genetic manipulation of *Haloferax* species” in Archaea: Methods and protocols. ed. Ferreira-Cerca S. (New York, NY: Springer US), 33–56. 10.1007/978-1-0716-2445-6_3 36125742

[ref14] Davis C. R. Johnson C. H. Robertson J. B. (2020). A bioluminescent reporter for the halophilic archaeon *Haloferax volcanii*. Extremophiles 24, 773–785. doi: 10.1007/s00792-020-01193-x, PMID: 32749548 PMC7462420

[ref15] de Silva R. T. Abdul-Halim M. F. Pittrich D. A. Brown H. J. Pohlschroder M. Duggin I. G. (2021). Improved growth and morphological plasticity of *Haloferax volcanii*. Microbiology 167:001012. doi: 10.1099/mic.0.001012, PMID: 33459585 PMC8131023

[ref16] Duggin I. G. Aylett C. H. Walsh J. C. Michie K. A. Wang Q. Turnbull L.. (2015). CetZ tubulin-like proteins control archaeal cell shape. Nature 519, 362–365. doi: 10.1038/nature13983, PMID: 25533961 PMC4369195

[ref17] Dyall-Smith M. (2009a). “Media recipes from the Thorsten Allers lab” in The halohandbook: Protocols for halobacterial genetics. ed. Dyall-Smith M. Available at: https://haloarchaea.com/wp-content/uploads/2018/10/Halohandbook_2009_v7.3mds.pdf

[ref18] Dyall-Smith M. (2009b). “Medium DBCM2, for square haloarchaea of Walsby (“*Haloquadratum walsbyi*”)” in The halohandbook: Protocols for halobacterial genetics. ed. Dyall-Smith M. Available at: https://haloarchaea.com/wp-content/uploads/2018/10/Halohandbook_2009_v7.3mds.pdf

[ref19] Dyall-Smith M. (2009c). “Trace element solution SL10” in The halohandbook: Protocols for halobacterial genetics. ed. Dyall-Smith M. Available at: https://haloarchaea.com/wp-content/uploads/2018/10/Halohandbook_2009_v7.3mds.pdf

[ref20] Dyall-Smith M. (2009d). “HF1 and HF2 - examples of lytic, head-tail haloviruses” in The halohandbook: Protocols for halobacterial genetics. ed. Dyall-Smith M. Available at: https://haloarchaea.com/wp-content/uploads/2018/10/Halohandbook_2009_v7.3mds.pdf

[ref21] Erdmann S. Tschitschko B. Zhong L. Raftery M. J. Cavicchioli R. (2017). A plasmid from an Antarctic haloarchaeon uses specialized membrane vesicles to disseminate and infect plasmid-free cells. Nat. Microbiol. 2, 1446–1455. doi: 10.1038/s41564-017-0009-2, PMID: 28827601

[ref22] Franzmann P. Stackebrandt E. Sanderson K. Volkman J. Cameron D. Stevenson P.. (1988). *Halobacterium lacusprofundi* sp. nov., a halophilic bacterium isolated from deep Lake, Antarctica. Syst. Appl. Microbiol. 11, 20–27. doi: 10.1016/S0723-2020(88)80044-4

[ref23] Hamm J. N. Erdmann S. Eloe-Fadrosh E. A. Angeloni A. Zhong L. Brownlee C.. (2019). Unexpected host dependency of Antarctic nanohaloarchaeota. Proc. Natl. Acad. Sci. U. S. A. 116, 14661–14670. doi: 10.1073/pnas.1905179116, PMID: 31253704 PMC6642349

[ref24] Harrison C. Allers T. (2022). “Progress and challenges in archaeal genetic manipulation” in Archaea: Methods and protocols. ed. Ferreira-Cerca S. (New York, NY: Springer US), 25–31. 10.1007/978-1-0716-2445-6_2 36125741

[ref25] Hartman A. L. Norais C. Badger J. H. Delmas S. Haldenby S. Madupu R.. (2010). The complete genome sequence of *Haloferax volcanii* DS2, a model archaeon. PLoS One 5:e9605. doi: 10.1371/journal.pone.0009605, PMID: 20333302 PMC2841640

[ref26] Javier T.-C. Carmen Pire G. Rosa María M.-E. (2017). “Biocompounds from haloarchaea and their uses in biotechnology” in Archaea, Ch. 4. eds. Haitham S. Afef N. Kais G. (Rijeka: IntechOpen)

[ref27] Krupovič M. Forterre P. Bamford D. H. (2010). Comparative analysis of the mosaic genomes of tailed archaeal viruses and proviruses suggests common themes for virion architecture and assembly with tailed viruses of bacteria. J. Mol. Biol. 397, 144–160. doi: 10.1016/j.jmb.2010.01.037, PMID: 20109464

[ref28] Lam W. L. Doolittle W. F. (1992). Mevinolin-resistant mutations identify a promoter and the gene for a eukaryote-like 3-hydroxy-3-methylglutaryl-coenzyme a reductase in the archaebacterium *Haloferax volcanii*. J. Biol. Chem. 267, 5829–5834. doi: 10.1016/S0021-9258(18)42628-2, PMID: 1556098

[ref29] Lestini R. Delpech F. Myllykallio H. (2015). DNA replication restart and cellular dynamics of Hef helicase/nuclease protein in *Haloferax volcanii*. Biochimie 118, 254–263. doi: 10.1016/j.biochi.2015.07.022, PMID: 26215377

[ref30] Li J. Gao Y. Dong H. Sheng G.-P. (2022). Haloarchaea, excellent candidates for removing pollutants from hypersaline wastewater. Trends Biotechnol. 40, 226–239. doi: 10.1016/j.tibtech.2021.06.006, PMID: 34284891

[ref31] Li M. Wang R. Zhao D. Xiang H. (2014). Adaptation of the *Haloarcula hispanica* CRISPR-Cas system to a purified virus strictly requires a priming process. Nucleic Acids Res. 42, 2483–2492. doi: 10.1093/nar/gkt1154, PMID: 24265226 PMC3936756

[ref32] Liao Y. Ithurbide S. Evenhuis C. Löwe J. Duggin I. G. (2021). Cell division in the archaeon *Haloferax volcanii* relies on two FtsZ proteins with distinct functions in division ring assembly and constriction. Nat. Microbiol. 6, 594–605. doi: 10.1038/s41564-021-00894-z, PMID: 33903747 PMC7611241

[ref33] Liao Y. Williams T. J. Walsh J. C. Ji M. Poljak A. Curmi P. M. G.. (2016). Developing a genetic manipulation system for the Antarctic archaeon, *Halorubrum lacusprofundi*: Investigating acetamidase gene function. Sci. Rep. 6:34639. doi: 10.1038/srep34639, PMID: 27708407 PMC5052560

[ref34] Litchfield C. D. (2011). Potential for industrial products from the halophilic archaea. J. Ind. Microbiol. Biotechnol. 38, 1635–1647. doi: 10.1007/s10295-011-1021-9 21853327

[ref35] Liu Y. Demina T. A. Roux S. Aiewsakun P. Kazlauskas D. Simmonds P.. (2021). Diversity, taxonomy, and evolution of archaeal viruses of the class Caudoviricetes. PLoS Biol. 19:e3001442. doi: 10.1371/journal.pbio.3001442, PMID: 34752450 PMC8651126

[ref36] Liu H. Han J. Liu X. Zhou J. Xiang H. (2011). Development of *pyrF*-based gene knockout systems for genome-wide manipulation of the archaea *Haloferax mediterranei* and *Haloarcula hispanica*. J. Genet. Genomics 38, 261–269. doi: 10.1016/j.jgg.2011.05.003, PMID: 21703550

[ref37] Ludt K. Soppa J. (2019). Polyploidy in halophilic archaea: Regulation, evolutionary advantages, and gene conversion. Biochem. Soc. Trans. 47, 933–944. doi: 10.1042/bst20190256, PMID: 31189733

[ref38] Luk A. W. Williams T. J. Erdmann S. Papke R. T. Cavicchioli R. (2014). Viruses of haloarchaea. Life 4, 681–715. doi: 10.3390/life4040681, PMID: 25402735 PMC4284463

[ref39] Matsuda R. Suzuki S. Kurosawa N. (2022). Genetic study of four candidate holliday junction processing proteins in the thermophilic crenarchaeon *Sulfolobus acidocaldarius*. Int. J. Mol. Sci. 23:707. doi: 10.3390/ijms23020707, PMID: 35054893 PMC8775617

[ref40] Maurer S. Ludt K. Soppa J. (2018). Characterization of copy number control of two *Haloferax volcanii* replication origins using deletion mutants and haloarchaeal artificial chromosomes. J. Bacteriol. 200. doi: 10.1128/jb.00517-17, PMID: 29038254 PMC5717156

[ref41] McGenity T. J. Grant W. D. (1995). Transfer of *Halobacterium saccharovorum*, *Halobacterium sodomense, Halobacterium trapanicum* NRC 34021 and *Halobacterium lacusprofundi* to the genus *Halorubrum* gen. Nov., as *Halorubrum saccharovorum* comb. nov., *Halorubrum sodomense* comb. nov., *Halorubrum trapanicum* comb. nov., and *Halorubrum lacusprofundi* comb. nov. Syst. Appl. Microbiol. 18, 237–243. doi: 10.1016/S0723-2020(11)80394-2

[ref42] Mercier C. Thies D. Zhong L. Raftery M. J. Cavicchioli R. Erdmann S. (2022). In depth characterization of an archaeal virus-host system reveals numerous virus exclusion mechanisms. bioRxiv. doi: 10.1101/2022.10.18.512658 PMC10544981 37789858

[ref43] Nußbaum P. Gerstner M. Dingethal M. Erb C. Albers S.-V. (2021). The archaeal protein SepF is essential for cell division in *Haloferax volcanii*. Nat. Commun. 12:3469. doi: 10.1038/s41467-021-23686-9, PMID: 34103513 PMC8187382

[ref44] Nuttall S. D. Dyall-Smith M. L. (1993). HF1 and HF2: Novel bacteriophages of halophilic archaea. Virology 197, 678–684. doi: 10.1006/viro.1993.1643, PMID: 8249290

[ref45] Piatek P. Humphreys C. Raut M. P. Wright P. C. Simpson S. Köpke M.. (2022). Agr Quorum Sensing influences the Wood-Ljungdahl pathway in *Clostridium autoethanogenum*. Sci. Rep. 12:411. doi: 10.1038/s41598-021-03999-x, PMID: 35013405 PMC8748961

[ref46] Pietilä M. K. Roine E. Sencilo A. Bamford D. H. Oksanen H. M. (2016). *Pleolipoviridae*, a newly proposed family comprising archaeal pleomorphic viruses with single-stranded or double-stranded DNA genomes. Arch. Virol. 161, 249–256. doi: 10.1007/s00705-015-2613-x 26459284

[ref47] Pohlschroder M. Schulze S. (2019). Haloferax volcanii. Trends Microbiol. 27, 86–87. doi: 10.1016/j.tim.2018.10.004 30459094

[ref48] Porter K. Tang S.-L. Chen C.-P. Chiang P.-W. Hong M.-J. Dyall-Smith M. (2013). PH1: An archaeovirus of *Haloarcula hispanica* related to SH1 and HHIV-2. Archaea 2013:456318. doi: 10.1155/2013/456318, PMID: 23585730 PMC3622292

[ref49] Redder P. Linder P. (2012). New range of vectors with a stringent 5-fluoroorotic acid-based counterselection system for generating mutants by allelic replacement in *Staphylococcus aureus*. Appl. Environ. Microbiol. 78, 3846–3854. doi: 10.1128/AEM.00202-12, PMID: 22447609 PMC3346405

[ref50] Reuter C. J. Maupin-Furlow J. A. (2004). Analysis of proteasome-dependent proteolysis in *Haloferax volcanii* cells, using short-lived green fluorescent proteins. Appl. Environ. Microbiol. 70, 7530–7538. doi: 10.1128/AEM.70.12.7530-7538.2004, PMID: 15574956 PMC535168

[ref51] Singh A. Singh A. K. (2017). Haloarchaea: Worth exploring for their biotechnological potential. Biotechnol. Lett. 39, 1793–1800. doi: 10.1007/s10529-017-2434-y, PMID: 28900776

[ref52] Tschitschko B. Williams T. J. Allen M. A. Páez-Espino D. Kyrpides N. Zhong L.. (2015). Antarctic archaea–virus interactions: Metaproteome-led analysis of invasion, evasion and adaptation. ISME J. 9, 2094–2107. doi: 10.1038/ismej.2015.110, PMID: 26125682 PMC4542027

[ref53] van Wolferen M. Pulschen A. A. Baum B. Gribaldo S. Albers S. V. (2022). The cell biology of archaea. Nat. Microbiol. 7, 1744–1755. doi: 10.1038/s41564-022-01215-8, PMID: 36253512 PMC7613921

[ref54] Wendoloski D. Ferrer C. Dyall-Smith M. (2001). A new simvastatin (mevinolin)-resistance marker from *Haloarcula hispanica* and a new *Haloferax volcanii* strain cured of plasmid pHV2. The GenBank accession number for the sequence reported in this paper is AF123438. Microbiology 147, 959–964. doi: 10.1099/00221287-147-4-959 11283291

[ref55] Williams T. J. Liao Y. Ye J. Kuchel R. P. Poljak A. Raftery M. J.. (2017). Cold adaptation of the antarctic haloarchaea *Halohasta litchfieldiae* and *Halorubrum lacusprofundi*. Environ. Microbiol. 19, 2210–2227. doi: 10.1111/1462-2920.13705, PMID: 28217912

[ref56] Winter K. Born J. Pfeifer F. (2018). Interaction of haloarchaeal gas vesicle proteins determined by Split-GFP. Front. Microbiol. 9:1897. doi: 10.3389/fmicb.2018.01897, PMID: 30174663 PMC6107691

