# Supplementary material for: Improving the genetic system for Halorubrum lacusprofundi to allow in-frame deletions
Source: Front Microbiol. 2023 Mar 31;14:1095621. doi: 10.3389/fmicb.2023.1095621 (PMC10102395; doi:10.3389/fmicb.2023.1095621)
Supplement: Supplementary file 1 [file Data_Sheet_1.pdf]

Improving the genetic system for *Halorubrum lacusprofundi* to allow in-frame deletions

Laura Johanna Gebhard[1], Iain G. Duggin[2], Susanne Erdmann[1]*

[1]Max Planck Institute for Marine Microbiology, Archaeal Virology, Celsiusstrasse 1, 28359, Bremen, Germany.
[2]The Australian Institute for Microbiology and Infection, University of Technology Sydney, Sydney, New South Wales, Australia

* To whom correspondence should be addressed: Susanne Erdmann. Tel: +49 421 2028-7340; Email: serdmann@mpi-bremen.de

Supplementary Material

Table of contents

Supplementary Figures

Supplementary Figure 1: Attempt of a Δ*pyrE2* deletion in *Hrr. lacusprofundi* ACAM34_UNSW using a PCR product of the Δ*pyrE2* construct.

Supplementary Figure 2: Δ*pyrE2* deletion in ACAM34_UNSW using pTA131_Δ*pyrE2.*

Supplementary Figure 3: Δ*pyrE2* deletion in ACAM34_UNSW using pTA131_Δ*pyrE2_hmgA.*

Supplementary Figure 4: Light microscopy images from wild type and mutant *Hrr. lacusprofundi* cultures.

Supplementary Figure 5: Δ*pyrE2* deletion using pTA131_Δ*pyrE2* in ACAM34_DSMZ.

Supplementary Figure 6: Transformation of pTA131_*hmgA*_Δ*trpA* into ACAM34_UNSW strains.

Supplementary Figure 7: PCR using genomic primers targeting *trpA* on ACAM34_UNSWΔ*pyrE2* transformants positive for pTA131_*hmgA*_Δ*trpA.*

Supplementary Figure 8: Pop-out of G4 of ACAM34_UNSWΔ*pyrE2* transformants positive for pTA131_*hmgA*_Δ*trpA.*

Supplementary Figure 9: Pop-out of G8 of ACAM34_UNSWΔ*pyrE2* transformants positive for pTA131_*hmgA*_Δ*trpA.*

Supplementary Figure 10: Test for tryptophan auxotrophy of ACAM34_UNSWΔ*pyrE2*Δ*trpA* strains.

Supplementary Figure 11: PCR using primers targeting *Hlac_2746* in ACAM34_UNSWΔ*pyrE2*Δ*trpA* S2 transformants**.**

Supplementary Figure 12: Screening of G9 of pTA132_Δ*Hlac_2746* transformants of ACAM34_UNSW*ΔpyrE2*Δ*trpA* S2 for successful pop-out.

Supplementary Figure 13: Screening of G8 of pTA131_hmgA_Δ*Hlac_2746* transformants of ACAM34_UNSWΔ*pyrE2* for successful pop-out.

Supplementary Tables

Supplementary Table 1: Plasmids used in this study
Supplementary Table 2: Primers used in this study

References

Supplementary Figures

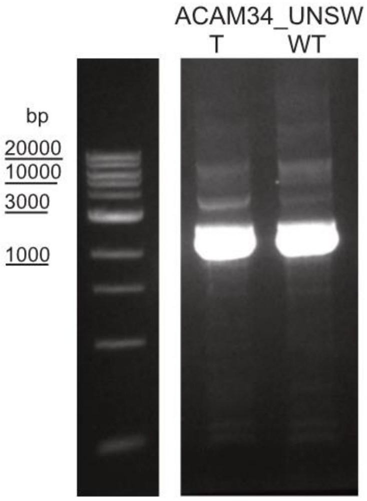

Supplementary Figure 1. Attempt of a Δ*pyrE2* deletion in *Hrr. lacusprofundi* ACAM34_UNSW using a PCR product of the Δ*pyrE2* construct. PCR with primers targeting the *pyrE2* gene region, on a liquid culture of cell material scratched from plates with a lawn of transformants. Samples from left to right: DNA Ladder (GeneRuler 1 kb Plus DNA Ladder, Thermo Scientific™)), (T) ACAM34_UNSW transformant culture and (WT) ACAM34_UNSW wild type control. DNA was separated on 1% agarose gels and stained with SYBR™ Safe.

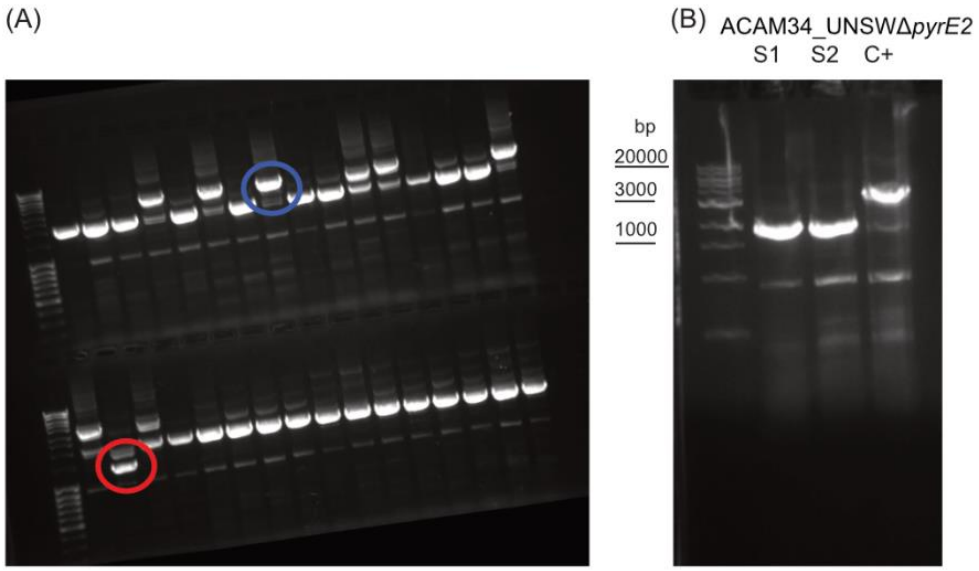

Supplementary Figure 2. Δ*pyrE2* deletion in ACAM34_UNSW using pTA131_Δ*pyrE2*.
Colony PCR with primers for the *pyrE2* gene region. (A) an exemplary colony with a transposon insertion is marked with a blue circle and the only colony with a deletion (ACAM34_UNSW_Δ*pyrE2*) is marked with a red circle. (B) two clones (S1 and S2 (for S2 see Supplementary Figure 3)) of ACAM34_UNSWΔ*pyrE2* after re-streaking together with a wild type ACAM_UNSW control (C+). First lane from the left in all panels: DNA marker (GeneRuler 1 kb Plus DNA Ladder, Thermo Scientific™). DNA was separated on 1% agarose gels and stained with SYBR™ Safe.

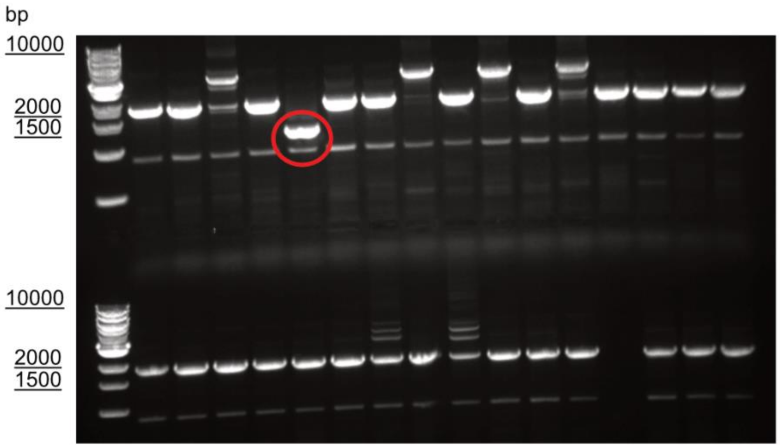

Supplementary Figure 3. Δ*pyrE2* deletion in ACAM34_UNSW using pTA131_Δ*pyrE2_hmgA*. Colony PCR with primers targeting the *pyrE2* gene on pTA131_Δ*pyrE2_hmgA* transformants. One colony showed a *pyrE2* gene of reduced size (labelled with a red circle) and was subsequently sequenced (ACAM34_UNSWΔ*pyrE2* S2). First lane from the left in all panels: DNA marker (GeneRuler 1 kb Plus DNA Ladder, Thermo Scientific™). DNA was separated on 1% agarose gels and stained with SYBR™ Safe.

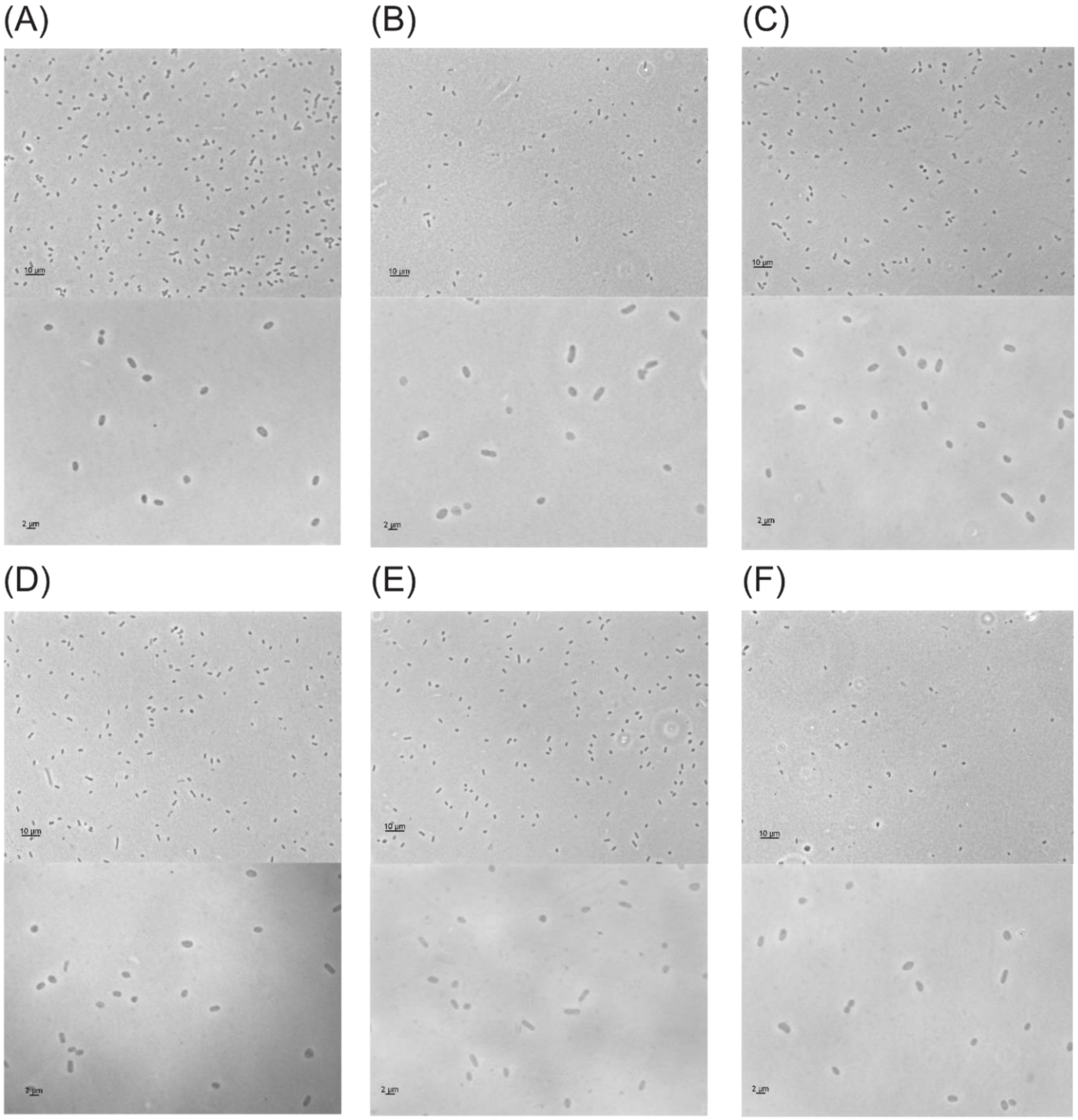

Supplementary Figure 4: Light microscopy images from wild type and mutant *Hrr. lacusprofundi* cultures. Cells imaged during mid-exponential growth (OD_{600}: 0.55-0.65) on agar coated slides (4%). (A-C) three biological replicates of ACAM34_UNSW cultures, (D-F) three biological replicates of ACAM34_UNSWΔ*pyrE2* cultures. Top images at 400x magnification, scale bars represent 10 µm. Bottom images at 1000x magnification, scale bars represent 2 µm.

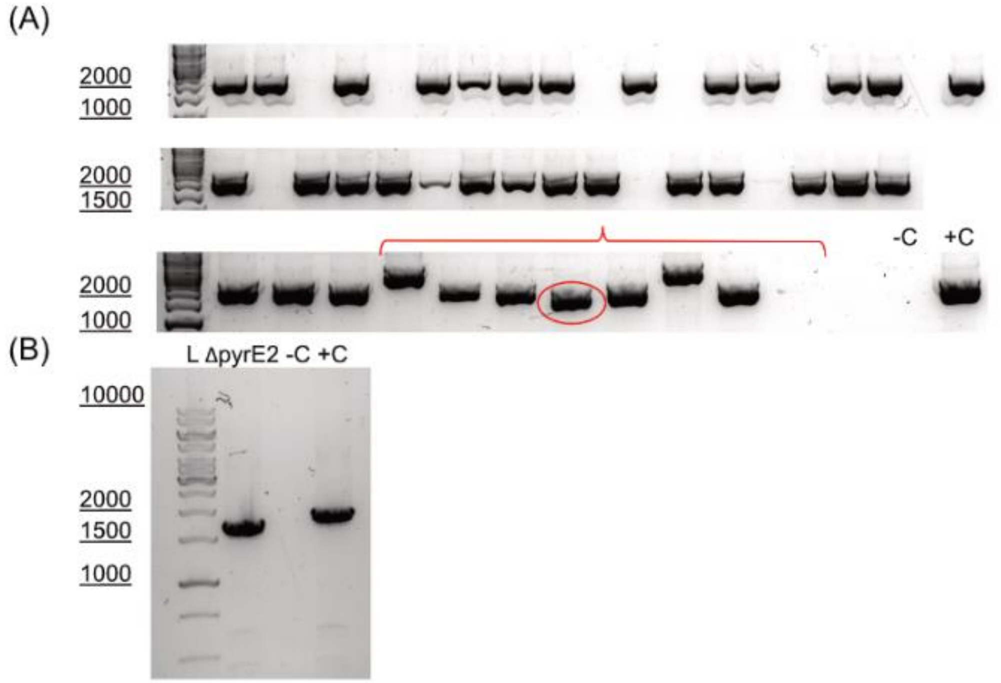

Supplementary Figure 5: Δ*pyrE2* deletion using pTA131_Δ*pyrE2* in ACAM34_DSMZ. (A) Colony PCR with primers targeting the *pyrE2* gene on pTA131_Δ*pyrE2* transformants and on colonies from a H_2O control transformation (marked with a red bracket). A colony with a PCR product that was slightly reduced in size (labelled with a red circle) was chosen for further characterization (ACAM_DSMZΔ*pyrE2*). (B) PCR with primers targeting the *pyrE2* gene on ACAM34_DSMZΔ*pyrE2* after propagation of the colony in liquid media. ACAM34_DSMZ wild type served as control (+C). First lane from the left in all panels: DNA marker (GeneRuler 1 kb Plus DNA Ladder, Thermo Scientific™). DNA was separated on 1% agarose gels and stained with SYBR™ Safe.

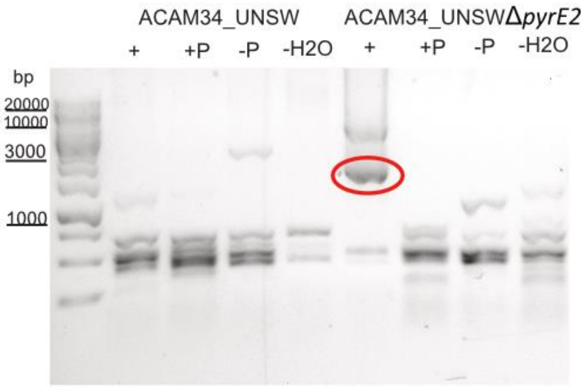

Supplementary Figure 6: Transformation of pTA131_*hmgA*_Δ*trpA* into ACAM34_UNSW strains. PCR with primers targeting the *hmgA* gene on pTA131_*hmgA*_Δ*trpA* on liquid cultures of transformants of ACAM34_UNSW and ACAM34_UNSWΔ*pyrE2*. (+) pTA131_*hmgA*_Δ*trpA*, (+P) pTA131_*hmgA* control, (-P) pTA131 control, (-H_2O) H_2O control. pTA131_*hmgA*_Δ*trpA* could only be detected in ACAM34_UNSWΔ*pyrE2* (red circle). Remaining bands are products of unspecific primer binding. First lane from the left: DNA marker (GeneRuler 1 kb Plus DNA Ladder, Thermo Scientific™). DNA was separated on 1% agarose gels and stained with SYBR™ Safe.

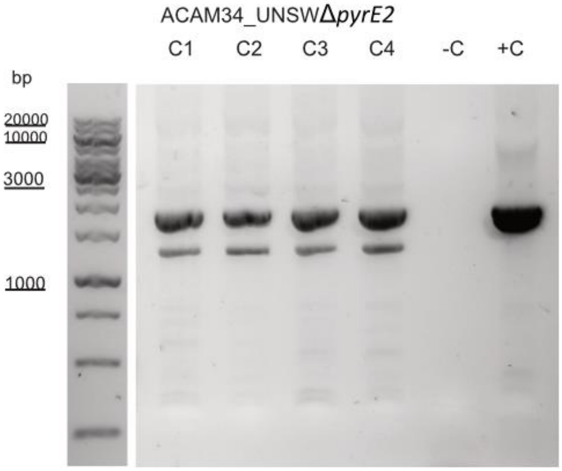

Supplementary Figure 7: PCR using genomic primers targeting *trpA* on ACAM34_UNSWΔ*pyrE2* transformants positive for pTA131_*hmgA*_Δ*trpA*. PCR with primers targeting the *trpA* gene on pTA131_*hmgA*_Δ*trpA* transformants of ACAM34_UNSWΔ*pyrE2*. (C1-C4) Colonies 1-4, (-C) negative control, (+C) positive control. All transformants show a mixture of wild type and mutant *trpA*. First lane from the left: DNA marker (GeneRuler 1 kb Plus DNA Ladder, Thermo Scientific™). DNA was separated on 1% agarose gels and stained with SYBR™ Safe.

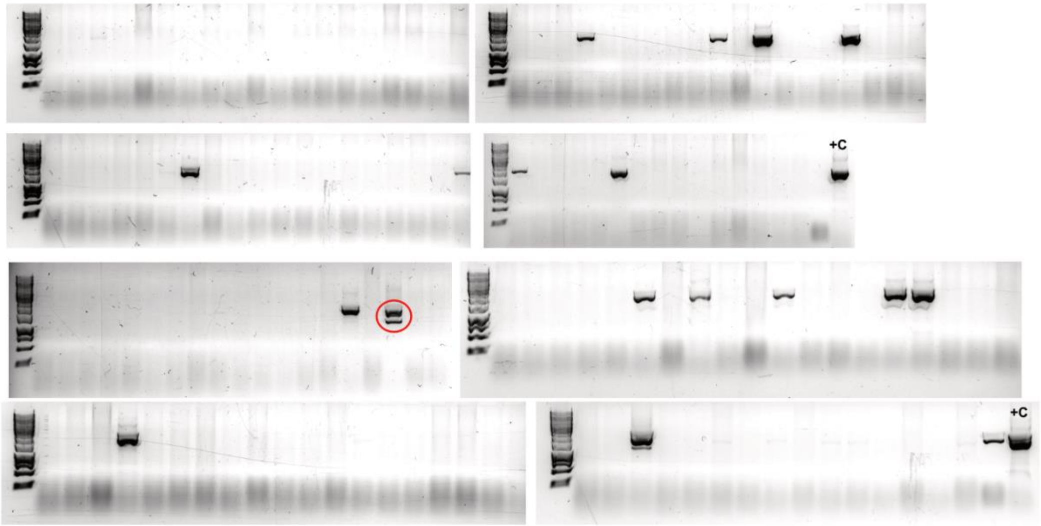

Supplementary Figure 8: Pop-out of G4 of ACAM34_UNSWΔ*pyrE2* transformants positive for pTA131_*hmgA*_Δ*trpA*. PCR using genomic primers targeting *trpA* on colonies of pTA131_*hmgA*_Δ*trpA* transformant ACAM34_UNSWΔ*pyrE2* after pop-out of G4. (+C) positive control. Lanes with no signal are colonies that still include the plasmid where the pop-out was not successful. Only one colony showed a mixture of wild type and mutant *trpA* (red circle). First lane from the left in all panels: DNA marker (GeneRuler 1 kb Plus DNA Ladder, Thermo Scientific™). DNA was separated on 1% agarose gels and stained with SYBR™ Safe.

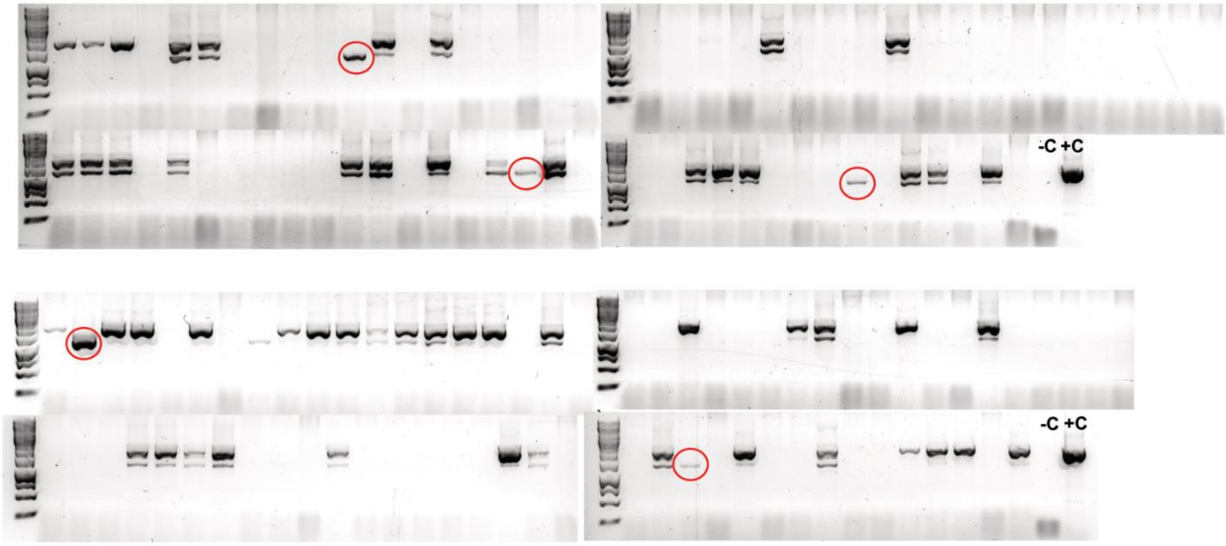

Supplementary Figure 9: Pop-out of G4 of ACAM34_UNSWΔ*pyrE2* transformants positive for pTA131_*hmgA*_Δ*trpA*. PCR using genomic primers targeting *trpA* on colonies of pTA131_*hmgA*_Δ*trpA* transformant ACAM34_UNSWΔ*pyrE2* after pop-out of G8. (+C) positive control, (-C) negative control. Lanes with no signal are colonies that still include the plasmid where the pop-out was not successful. The majority of colonies showed a mixture of wild type and mutant *trpA,* only five appeared to have a clean deletion (red circles). First lane from the left in all panels: DNA marker (GeneRuler 1 kb Plus DNA Ladder, Thermo Scientific™). DNA was separated on 1% agarose gels and stained with SYBR™ Safe.

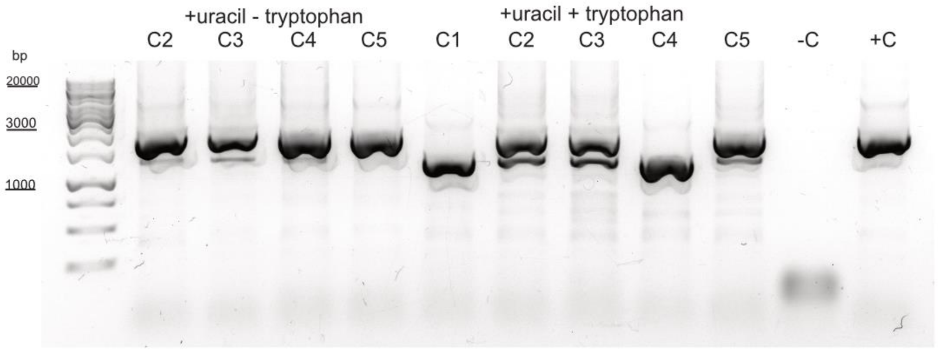

Supplementary Figure 10: Test for tryptophan auxotrophy of ACAM34_UNSWΔ*pyrE2*Δ*trpA* strains. PCR using genomic primers targeting *trpA* on 5 potential ACAM34_UNSWΔ*pyrE2*Δ*trpA* clones (+C) positive control, (-C) negative control. (C1-C5) Colonies 1-5. C2-5 converted to wild type in media without tryptophan, indicating that they had some remaining wild type copies of *trpA*. C1 was not able to grow in in media without tryptophan and shows a clean deletion in the *trpA* gene. First lane from the left: DNA marker (GeneRuler 1 kb Plus DNA Ladder, Thermo Scientific™). DNA was separated on 1% agarose gels and stained with SYBR™ Safe.

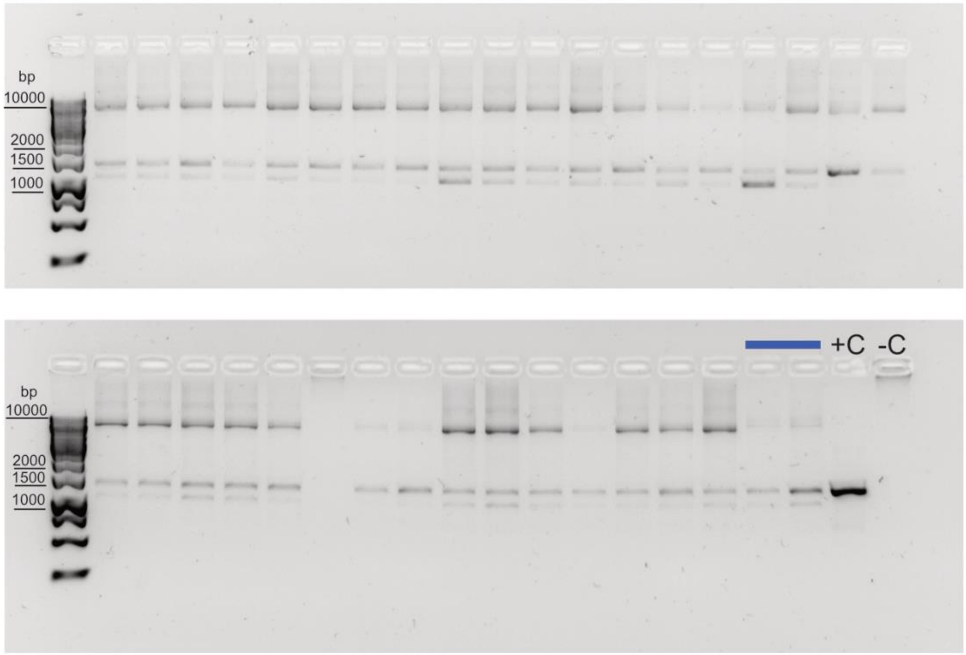

Supplementary Figure 11: PCR using primers targeting *Hlac_2746* in ACAM34_UNSWΔ*pyrE2*Δ*trpA* S2 transformants. PCR using genomic primers targeting the predicted GTPase (*Hlac_2746*) on 36 potential ACAM34_UNSWΔ*pyrE2*Δ*trpA* pTA132_*ΔHlac_2746* transformants with 6 min elongation (+C) positive control wild type ACAM_UNSW genomic DNA, (-C) negative control (H_2O). (C1-C34) colonies from G4 after successive culturing in selective media. (C35-36, marked with blue bar) liquid cultures (G5) after cell material was scratched from G4 plates. Bands at around 1500 bp indicate wild type Hlac_2746 presence, larger bands near 10,000 bp indicate the presence of the knockout plasmid integrated into the genome (successful pop-in). Smaller than WT bands could indicate already mutated *Hlac_2746*. First lane from the left in both gels: DNA marker (GeneRuler 1 kb Plus DNA Ladder, Thermo Scientific™). DNA was separated on 1% agarose gels and stained with SYBR™ Safe.

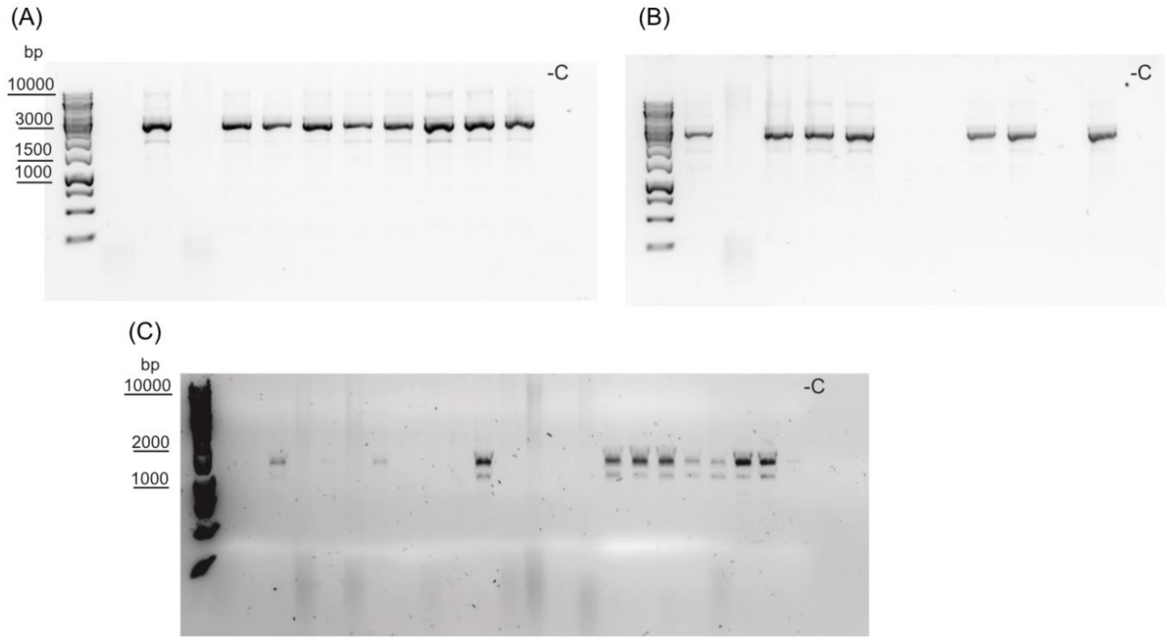

Supplementary Figure 12: Screening of G9 of pTA132_Δ*Hlac_2746* transformants of ACAM34_UNSWΔ*pyrE2* Δ*trpA* S2 for successful pop-out. PCRs targeting the predicted GTPase (Hlac_2746) on 22 potential ACAM34_UNSWΔ*pyrE2*Δ*trpA*Δ*Hlac_2746* clones. (A-B) PCR with forward primer binding the genome outside of *Hlac_2746* and the reverse primer binding pTA132. (-C) negative controls (H_2O), (A) C1-11 and (B) C12-22 colonies picked from G9 plates. Amplified bands indicate plasmid presence in the genome and an unsuccessful pop-out. (C) PCR using genomic primers targeting *Hlac_2746* on C1-22, (-C) negative control (H_2O). Amplified fragments indicate either wild type or mutated *Hlac_2746*, while no amplification indicates plasmid presence (unsuccessful pop-out). First lane from the left: DNA marker (GeneRuler 1 kb Plus DNA Ladder, Thermo Scientific™). DNA was separated on 1% agarose gels and stained with SYBR™ Safe.

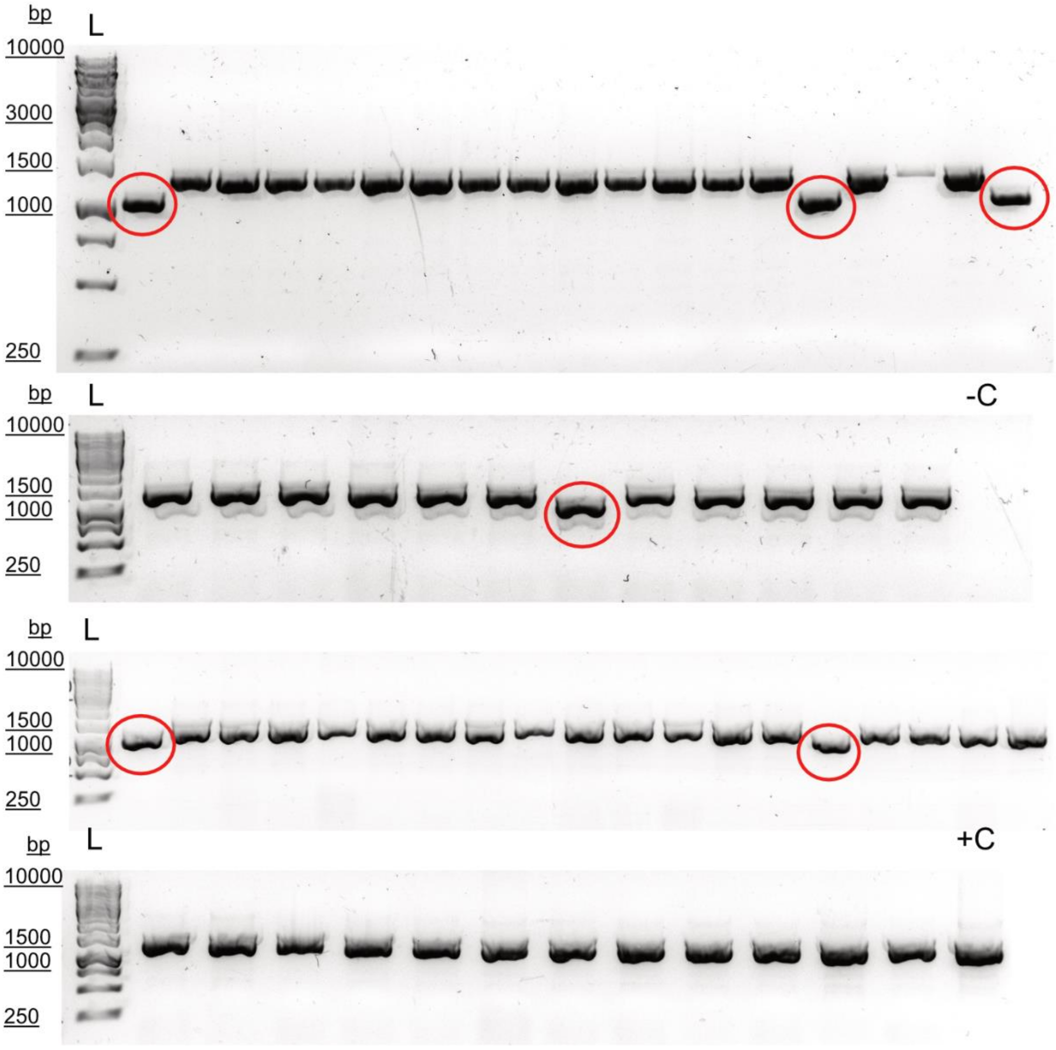

Supplementary Figure 13: Screening of G8 of pTA131_*hmgA*_Δ*Hlac_2746* transformants of ACAM34_UNSWΔ*pyrE2* for successful pop-out. PCR targeting the predicted GTPase (*Hlac_2746*) on 62 potential ACAM34_UNSWΔ*pyrE2*Δ*Hlac_2746* clones. Clones with PCR signals for mutated *Hlac_2746* are marked with red circles. (-C) negative control with ddH_2O, (+C) positive control with wild type ACAM34_UNSW genomic DNA. First lane from the left: DNA marker (GeneRuler 1 kb Plus DNA Ladder, Thermo Scientific™). DNA was separated on 1% agarose gels and stained with SYBR™ Safe.

Supplementary Tables

Supplementary Table 1: Plasmids used in this study

**plasmid**	**Relevant properties**	**Reference or source**
pTA131	pBluescript II derived, non-replicative *H. volcanii* plasmid containing *pyrE2*	[1]
pTA131_Δ*pyrE2*	pTA131 with *Hind*III and *Bam*HI fragment containing *pyrE2* nonfunctional construct for gene inactivation	This study
pTA131_*hmgA*	pTA131 with *hmgA* from pJWID1 inserted with *Xba*I and *Not*I to confer pravastatin resistance	This study
pTA131_*ΔpyrE2_ hmgA*	pTA131_Δ*pyrE2* with *hmgA* from pJWID1 inserted with *Xba*I and *Not*I to confer pravastatin resistance	This study
pTA131_*hmgA_ΔtrpA*	pTA131_*hmgA* with *Hind*III and *Bam*HI fragment containing *trpA* nonfunctional construct for gene inactivation and a functional *pyrE2* gene for complementation	This study
pTA131_*hmgA_ΔHlac_2746*	pTA131_*hmgA* with *Hind*III and *Bam*HI fragment containing Hlac_2746 (GTPase) nonfunctional construct for gene inactivation and a functional *pyrE2* gene for complementation	This study
pTA132	pBluescript II derived, non-replicative *H. volcanii* plasmid containing *trpA*	[1]
pTA132_ *ΔHlac_2746*	pTA132 with *Hind*III and *Bam*HI fragment containing GTPase (Hlac_2746) nonfunctional construct for gene inactivation	This study
pJWID1	*H.volcanii* shuttle vector containing *pyrE2*, *hdrB*, *hmgA*, *bla*, SmRS-GFP and pHV2 origin of replication	[2]

Supplementary Table 2: Primers used in this study

Name	Sequence 5' -> 3'	Description	product length (bp)	annealing temperature Q5 polymerase (°C)	Restriction Sites (if applicable)
Pyr_USF	CCGGCCAAGCTTGTGAATATGTTGATCCGAGCGTG	Forward primer to amplify upstream region of *Hrr. lacusprofundi pyrE2* gene Hlac_0584 for knock out	686	58 -> 72	*Hind*III
Pyr_USR	GAATTCGCCGCCCGAAGATCTGTACTTGTCGACGTAGTAGTCGC	Reverse primer to amplify upstream region of *Hrr. lacusprofundi pyrE2* gene Hlac_0584 for knock out	686	58 -> 72	*Eco*RI & *Bgl*II
Pyr_DSF	AGATCTTCGGGCGGCGAATTCGACGGAATTGCTGGCGGAACGC	Forward primer to amplify downstream region of *Hrr. lacusprofundi pyrE2* gene Hlac_0584 for knock out	637	58 -> 72	*Eco*RI & *Bgl*II
Pyr_DSR	CCGGCCGGATCCCTACGTCATCGCGACGCAGAAC	Reverse primer to amplify downstream region of *Hrr. lacusprofundi pyrE2* gene Hlac_0584 for knock out	637	58 -> 72	*Bam*HI
Seq_PyrF	GATGAACGTGCGCCGAGAGAC	Forward genomic sequencing primer binding upstream of *Hrr. lacusprofundi pyrE2* gene Hlac_0584	1923 (WT)	70	
Seq_PyrR	CGACCTCCTTCCCAGCGACGTG	Reverse genomic sequencing primer binding downstream of *Hrr. lacusprofundi pyrE2* gene Hlac_0584	1923 (WT)	70	
prav_F	CTCATCTAGAGTGCCTAATGAGTGAGCTAAC	Forward primer for cloning of *hmgA* gene (*Haloferax*) into pTA131	1550	58 -> 72	*Xba*I
prav_R	CTCAGCGGCCGCACTCTGAACCTATGAATCGAG	Reverse primer for cloning of *hmgA* gene (*Haloferax*) into pTA131	1550	58 -> 72	*Not*I
1273UPF	CCGGCCAAGCTTGGAAGAGTTCCACGAGGAAC	Forward primer to amplify upstream region of *Hrr. lacusprofundi trpA* gene Hlac_1273 for knock out	330	64 -> 72	*Hind*III
1273UPR	GAATTCGCCGCCCGAAGATCTCGAGAACGGGAGTCCGAGT	Reverse primer to amplify upstream region of *Hrr. lacusprofundi trpA* gene Hlac_1273	330	64 -> 72	*Eco*RI & *Bgl*II

1273DSF	AGATCTTCGGGCGGCGAATTCGTCGAGGACGACCTCTCGAC	Forward primer to amplify downstream region of *Hrr. lacusprofundi trpA* gene Hlac_1273	650	66 -> 72	*Eco*RI & *Bgl*II
1273DSR	CCGGCCGGATCCGTCCGAGCGATTCAGGATCTG	Forward primer to amplify downstream region of *Hrr. lacusprofundi trpA* gene Hlac_1273	650	66 -> 72	*Bam*HI
1273seqF_v2	CAGATCATGGAGAGCCACTCG	Forward genomic sequencing primer binding upstream of *Hrr. lacusprofundi trpA* gene Hlac_1273	1870 (WT)	69	
1273seqR_v2	GTAGCCGGTTTTCACCACGTC	Reverse genomic sequencing primer binding downstream of *Hrr. lacusprofundi trpA* gene Hlac_1273	1870 (WT)	69	
Hlac_2746UF	CCGGCCAAGCTTCTGACGCAGTTCTTAAGTG	Forward primer to amplify upstream region of *Hrr. lacusprofundi GTPase* gene Hlac_2746 for knock out	321	61 -> 72	*Hind*III
Hlac_2746UR	GAATTCGCCGCCCGAAGATCTCGAGTTTCGTGTGGAATGTG	Reverse primer to amplify upstream region of *Hrr. lacusprofundi GTPase* gene Hlac_2746 for knock out	321	61 -> 72	*Eco*RI & *Bgl*II
Hlac_2746DF	AGATCTTCGGGCGGCGAATTCGATGCTCATCGGGATCATC	Forward primer to amplify downstream region of *Hrr. lacusprofundi GTPase* gene Hlac_2746 for knock out	525	66 -> 72	*Eco*RI & *Bgl*II
Hlac_2746DR	CCGGCCGGATCCGAGCTTCTGGGTCGAGTTG	Reverse primer to amplify downstream region of *Hrr. lacusprofundi GTPase* gene Hlac_2746 for knock out	525	66 -> 72	*Bam*HI
Hlac_2746KOF	CGAATAAGTAAACAACCGATATC	Forward genomic sequencing primer binding upstream of *Hrr. lacusprofundi GTPase* gene Hlac_2746	1270	59	
Hlac_2746KOR	GGTGTGGCATTTACTTACG	Reverse genomic sequencing primer binding downstream of *Hrr. lacusprofundi GTPase* gene Hlac_2746	1270	59	
M13/pUC_R	AGCGGATAACAATTTCACACAGG	Reverse plasmid screening primer for pUC/pTA plasmids	ca. 200	66	

References

1. Allers, T., Ngo, H.-P., Mevarech, M., & Lloyd, R. G. (2004). Development of Additional Selectable Markers for the Halophilic Archaeon Haloferax volcanii Based on the leuB and trpA Genes. Applied and Environmental Microbiology, 70(2), 943-953. doi:10.1128/aem.70.2.943-953.2004

2. Liao, Y., Williams, T. J., Walsh, J. C., Ji, M., Poljak, A., Curmi, P. M. G., . . . Cavicchioli, R. (2016). Developing a genetic manipulation system for the Antarctic archaeon, Halorubrum lacusprofundi: investigating acetamidase gene function. Scientific Reports, 6, 34639. doi:10.1038/srep34639
